# Transcriptional Structure of Petunia Clock in Leaves and Petals

**DOI:** 10.3390/genes10110860

**Published:** 2019-10-30

**Authors:** Marta I. Terry, Marta Carrera-Alesina, Julia Weiss, Marcos Egea-Cortines

**Affiliations:** Genética Molecular, Instituto de Biotecnología Vegetal, Edificio I+D+I, Plaza del Hospital s/n, Universidad Politécnica de Cartagena, 30202 Cartagena, Spain; marta.terry@edu.upct.es (M.I.T.); julia.weiss@upct.es (J.W.)

**Keywords:** flower development, circadian clock, entrainment of circadian rhythm, petal, Solanaceae, tissue specific, transcriptional noise

## Abstract

The plant circadian clock coordinates environmental signals with internal processes including secondary metabolism, growth, flowering, and volatile emission. Plant tissues are specialized in different functions, and petals conceal the sexual organs while attracting pollinators. Here we analyzed the transcriptional structure of the petunia (*Petunia x hybrida*) circadian clock in leaves and petals. We recorded the expression of 13 clock genes in petunia under light:dark (LD) and constant darkness (DD). Under light:dark conditions, clock genes reached maximum expression during the light phase in leaves and the dark period in petals. Under free running conditions of constant darkness, maximum expression was delayed, especially in petals. Interestingly, the rhythmic expression pattern of *PhLHY* persisted in leaves and petals in LD and DD. Gene expression variability differed among leaves and petals, time of day and photoperiod. The transcriptional noise was higher especially in leaves under constant darkness. We found that *PhPRR7*, *PhPRR5*, and *PhGI* paralogs showed changes in gene structure including exon number and deletions of CCT domain of the PRR family. Our results revealed that petunia petals presented a specialized clock.

## 1. Introduction

Organisms, from bacteria to human beings, are subjected to periodic oscillations in the environment due the planet rotation around its axis. Circadian clocks are a complex set of genes allowing organisms to anticipate and adapt to daily environmental variations. In plants, the circadian clock is a network of interlocked loops comprising transcriptional, translational, and posttranslational coordination [1]. Circadian processes have been studied in plants for a long period of time (see McClung for a historical overview [2]). Most molecular studies have been done in *Arabidopsis thaliana*. The Arabidopsis core clock is formed by several genes. Two MYB transcription factors CIRCADIAN CLOCK ASSOCIATED 1 (*CCA1*), LATE ELONGATED HYPOCOTYL (*LHY*)*,* and the pseudo response regulator TIMING OF CAB EXPRESSION (*TOC1*) form the so-called core clock. Later studies found other clock components including the PSEUDO-RESPONSE REGULATOR gene family (*PRR*), out of which PRR3, PRR5, PRR7, and PRR9 are clock genes, and the Evening Complex (EC), which is formed by the EARLY FLOWERING 3 (*ELF3*), EARLY FLOWERING 4 (*ELF4*)*,* and LUX ARRHYTMO (*LUX*) proteins. In addition, other genes playing a key role and considered part of the clock include the protein with blue light reception capacity ZEITLUPE (*ZTL*) and the single copy gene GIGANTEA (*GI*). The various models developed are based on mutually repressing genes and a set of activating genes coded by the REVEILLE MYB transcription factors [3]. Every new discovery has added a level of complexity and new interpretation of the circadian clock model [4].

Two aspects emerge from comparative genomics with lower organisms and within higher plants. First the core clock components identified in the picoeukaryote *Ostreococcus* comprise a *MYB* gene homolog to *LHY* and a *PRR* gene similar to *TOC1* [5]. There is an additional blue-light receptor component with histidine kinase activity and circadian clock effects [6]. Therefore, basic clocks may be found with two or maybe three components that function via transcriptional control. A second aspect is that the fine tuning of the different clock modules is based to a large extent on protein–protein interactions. As protein complexes require certain stoichiometries to maintain their function they are target of genetic constraints in terms of gene dosages and are especially sensitive to gene duplications. Duplicated genes follow four paths including gene loss, maintenance of redundancy, subfunctionalization, or neofunctionalization [7]. Plant genomes have been subject to genome duplications and in some cases, followed by non-random elimination of duplicated genes [8,9]. In *Brassica*, polyploidization events have involved subsequent gene loss but with a preferential retention of circadian clock genes as compared to house-keeping genes, supporting a gene dosage sensitivity model [10]. 

The genomes of the garden petunia and its ancestors *Petunia axillaris* and *P. integrifolia* have been recently sequenced [11]. Petunia forms an early branching in the Solanaceae clade departing from *Solanum lycopersicon, S. tuberosum, Nicotiana* spp. and *Capsicum* spp. that have a chromosome number of n = 12. Petunia has n = 7 and this, together with a high activity of transposition, may have shaped a somewhat different genome evolution. Petunia shares a paleohexaplodization specific to the Solanaceae. A comprehensive analysis of the circadian clock genes found in the *Petunia* genomes shows that there is a set of genes that has remained as single copy. These include the petunia orthologs for *PRR9*, *PRR3*, *TOC1*, and *LHY*. In contrast, other genes are present in two to four copies, *PRR7*, *PRR5*, *GI*, *ELF3*, or *ELF4* [11]. Altogether these data indicate a possible departure of the circadian clock network from the one known in Arabidopsis, and suggests the evolution of the clock at different levels including gene structure, expression pattern, and genetic functions.

The bulk of work on plant circadian rhythms has been done in Arabidopsis using leaf tissue and seedlings. Like in animals, there is important evidence that the circadian clock expression network differs between different organs. The current view is that the shoot apical meristem may work as a center of coordination [12], and leaves and roots differ in the regulatory network, as a result of differences in light inputs [13,14]. 

Petal development starts with the activation of the so-called B function genes in both gymnosperms and angiosperms [15]. The initial transcriptional activation is followed at early stages by an autoregulatory positive regulation of the MADS-box genes controlling petal morphogenesis in *Antirrhinum*, Arabidopsis, and petunia [16,17,18,19,20,21]. Once organ identity is established and right after anthesis, there is a transcriptional reprogramming [22]. Furthermore, in sympetalous flowers with petals forming a tube and a limb, both parts of the flower appear to have different functions and transcriptional control [23,24]. The petal function after anthesis includes concealing the sexual organs and attracting pollinators. The lifespan of a flower is relatively short with most flowers surviving two to five days after anthesis. After anthesis, metabolism and scent emission changes rapidly [25,26]. Flowers enter rapid senescence upon pollination as a result of ethylene release [27,28,29].

Floral scent release depends on petal development in a quantitative way [22], and is circadian regulated in monocots and dicots such as *Antirrhinum*, *Narcissus*, rose, or garden petunia [30,31,32,33,34]. Most flowers analyzed emit scent preferentially during the day or during the night. The *LHY* and *ZTL* orthologs control scent emission in *Petunia*, *Nicotiana attenuata*, and *Antirrhinum majus* [35,36,37,38]. The Solanaceae Petunia and Nicotiana emit higher quantities during the night, indicating an identity and circadian component controlling this trait [34,39]. 

In the current work, we have addressed the structure of the petunia circadian clock from three different perspectives: gene structure, transcript abundance, and gene expression variability. The gene structure diverges as *PRR* paralogs have different intron numbers and *PhGI1* and *PhGI2* vary in the coding region. The transcriptional structure showed maximum expression during the day in leaves and during the dark in petals. This maximum tended to delay in both tissues under constant darkness conditions. We further identified opposite levels of transcriptional noise at dawn in leaves and dusk in petals. Our results reflect the evolution of the plant circadian clock at the structural and expression level and suggests an organ specific transcriptional structure of the plant circadian clock. 

## 2. Materials and Methods 

### 2.1. Plant Materials and Experiment Design 

We used the *Petunia hybrida* W115 or Mitchell for all the analyses. Plants were grown under greenhouse conditions until further use. Experiments under controlled conditions in growth chambers were performed as described [40], with the following modifications. For the control experiments, plants were adapted to light:dark growth chamber conditions for at least 1 week. Day:night (LD) conditions were matched with thermoperiods of 23 °C:18 °C during the light and dark periods. Zeitgeber time (ZT) was defined as ZT0 for light on and ZT12 for light off. Plants were transferred from LD cycle to a continuous dark cycle (DD) with the same temperature regimes. 

Flowers were marked before opening, and samples were taken at day 2–3 after anthesis. We used the petal limbs for all experimental procedures. We used young leaves with a length of 1.5–2.5 cm for all the experiments. Sampling of petal limbs and leaves was made every 3 h, starting at ZT0 and tissues were immediately frozen in liquid nitrogen. In the case of DD experiment, sampling also started at ZT0, during the first 24 h under continuous dark.

### 2.2. Phylogeny, Bioinformatics, and Data Analysis 

Previous studies showed that most genes of Petunia W115 can be assigned to *Petunia axillaris* [11]. We used clock genes sequences from *Arabidopsis thaliana* (https://www.arabidopsis.org/) to identify *P. axillaris* orthologs by blast in Sol Genomics Network (https://solgenomics.net/). Protein sequences from Petunia and other species were obtained from several databases including Sol Genomics Network (https://solgenomics.net/, Solanaceae), Snapdragon Genome Database (http://bioinfo.sibs.ac.cn/Am/, *Antirrhinum majus*) [41], Phytozome (https://phytozome.jgi.doe.gov/pz/portal.html) and GenBank (https://www.ncbi.nlm.nih.gov/genbank/) (Tables S1 and S2 for protein accessions and details). We used the corresponding predicted proteins to identify the intron-exon boundaries using Genewise [42]. The corresponding exon-intron boundaries were plotted using the exon-intron graphic maker (http://wormweb.org/exonintron). Protein alignment was performed with CLUSTALX [43]. Phylogenetic analysis was performed with MEGA X [44] using neighbor-joining method (NJ), JTT (Jones, Taylor and Thornton, [45]) as model of amino acid substitution and 500 bootstrap replicates. Phylogenetic tree of PSEUDO-RESPONSE REGULATORS (PRR) proteins was conducted by using the highly conserved domain pseudo-receiver (PR) [46] whereas phylogenetic tree of GIGANTEA (GI) proteins was performed by using full length proteins. Trees were visualized and annotated with “ggtree” [47] using R (R version 3.5.1), as well as the multiple sequence alignment. Protein domains were predicted using the web-based tool PROSITE [48], schematic proteins were plotted with the R package “drawProteins” [49].

Detection of rhythmic gene expression was performed using the non-parametric statistical algorithm “RAIN” [50]. We analyzed leaves and petals, under two light conditions, 12 h light/12 h dark (LD) and constant darkness (DD). To determine if the gene expression pattern of certain gene differed between tissues (leaves and petals) or light conditions (light:dark and constant darkness), we used an harmonic ANOVA (HANOVA) and to detect noise changes we used a scale test (HarmScaleTest) implemented in the R package “DODR” [51]. The HANOVA detects changes in amplitude and phases and HarmScaleTest uses a F-Test for variances to determine if the biological noise levels differed or not in two time series. We plotted the graphics with “ggplot2” [52]. 

### 2.3. Gene Expression Analysis by qPCR 

RNA was extracted from three biological replicates per time point of leaves and petals (flower tubes were excluded) using acid phenol [53]. Concentrations were measured using NanoDrop (Thermo-Fisher). Equal amounts of total RNA were used to obtain cDNA using Maxima kits (Thermo-Fisher).

PCR analysis was performed as described before [54], the following protocol was used for 40 cycles: 95 °C for 5 s, 60 °C for 20 s and 72 °C for 15 s (Clontech SYBR Green Master Mix and Mx3000P qPCR Systems, Agilent Technologies). Primers for circadian clock genes were designed using pcrEfficiency [55] (Table S3) and the following protocol was used for 40 cycles: 95 °C for 5 s, 60 °C for 20 s (55 °C for *PhGI1* and *PhGI2*) and 72 °C for 15 s. Samples were run in duplicate. Primer combinations were tested with genomic DNA from Mitchell and we found that all of them gave a single copy DNA on agarose gels. The endpoint PCR was further verified by melting point analysis where all primer combinations gave a single peak of melting (Figure S1). Normalized expression was calculated as described [56] and *ACTIN* (*PhACT*) was the internal control gene, a stable gene in circadian studies in petunia leaves and petals [37].

We determined the mean normalized expression. For each gene, tissue, and light condition, normalized expression was calculated as described previously [56], then the expression level for each time point was divided by the average expression across the time-course [57]. In addition, to analyze the gene expression variability, we calculated the coefficient of variation (CV) of every gene per time point, tissue and light:dark conditions. We also plotted the normalized expression of every biological replicate.

## 3. Results

### 3.1. The Duplicated PRR7, PRR5, and GI Diverge in Intron Number and Coding Sequence

**Figure 1:**
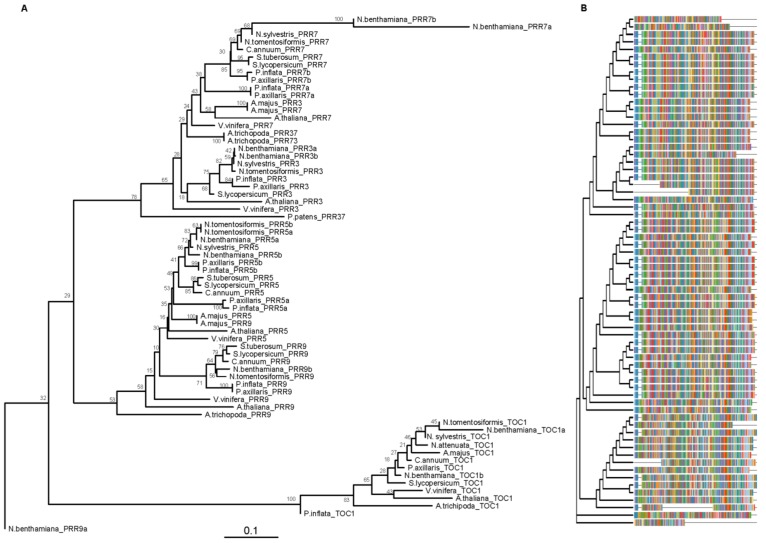
PSEUDO-RESPONSE REGULATORS (PRRs) phylogenetic tree. Pseudo-receiver (PR) amino acid sequences were aligned using CLUSTALX. Phylogenetic analysis was performed using MEGA X and trees were plotted with the library “ggtree” (R version 3.5.1). The phylogenetic tree was estimated using the neighbor-joining algorithm (NJ) JTT (Jones, Taylor and Thornton) as model of amino acid substitution. The tree shows the bootstrap percentage (from 500 replicates) next to branches. The tree was plotted to scale (**A**). The multiple sequence alignment is showed on the right side (**B**). This tree contains 69 sequences from 14 species. Species abbreviations: A.majus (*Antirrhinum majus*), A.thaliana (*Arabidopsis thaliana*), A.trichopoda (*Amborella trichopoda*), C.annuum (*Capsicum annuum*), N.attenuata (*Nicotiana attenuata*), N.benthamiana (*Nicotiana benthamiana),* N.sylvestris (*Nicotiana sylvestris*), N.tomentosiformis (*Nicotiana tomentosiformis*), P.axillaris (*Petunia axillaris*), P.inflata (*Petunia inflata*), P.patens (*Physcomitrella patens*), S.lycopersicum (*Solanum lycopersicum*), S.tuberosum (*Solanum tuberosum)* and V.vinifera (*Vitis vinifera*). Accessions are listed in Table S1.

We used the laboratory line *Petunia hybrida* W115, also known as Mitchell, which contains the circadian clock genes corresponding to *P. axillaris* [11] for a detailed analysis of the structure of the *PRR* and *GI* paralogs. Several genes forming the morning and evening loops of the circadian clock in petunia have undergone gene duplication. The genome of petunia has seven *PRR* genes as *PRR7* and *PRR5* are duplicated both in *P. axillaris* and *P. integrifolia* while Arabidopsis has the canonical set of five genes, *PRR1* or *TOC1, PRR3, PRR5*, *PRR7,* and *PRR9* involved in circadian regulation [11]. We reconstructed a phylogenetic tree of *PRR* genes, by using the conserved domain pseudo-receiver (PR), of Solanaceae and Arabidopsis (Table S1) in order to deduce the evolutionary relationships of the duplicated genes. As found previously for other Angiosperms, the *PRR* genes of Solanaceae form three major clades: the *TOC1/PRR1* clade, the *PRR7/3* clade, and the *PRR9*/*5* clade (Figure 1) [58]. The *PRR5a* genes of *P. axillaris*, *P. integrifolia* are closer to the Arabidopsis *AtPRR5* while the rest of the *PRR* genes of Solanaceae, including the *PRR5b,* form an additional subclade. This topology indicates that the *PRRa* paralogs may be an ancestral form and the *PRRb* may have been formed later and retained, in some cases as single copy genes. The *PRR7* genes also showed a similar topology where *PaxiNPRR7a* and *PinfS6PRR7a* are closer to the Arabidopsis gene than the single copy genes of the rest of the Solanaceae, and the *PRR7b* paralogs. This topology is also seen in petunia *PRR9*, *PRR3,* and *TOC1* that are somewhat between the Arabidopsis gene and the rest of the Solanaceae, according to the early departure of *Petunia* from the rest of the family [11].

We found that the gene models for *PhPRR5a* and *PhPRR5b* differ in the number of exons comprising the coding region as *PhPRR5a* has seven and *PhPRR5b* eight exons (Figure S2). The gene model in Arabidopsis comprises six exons in AT5G24470 (*AtPRR5*), indicating that changes in intron-exon structure has occurred in the evolution of the *PRR* family. The number of exons also differed between *PhPRR7a* with eight exons while *PhPRR7b* had seven exons. The Arabidopsis AT5G02810 *AtPRR7* has nine exons out of which eight correspond to coding region, thus coinciding with the phylogenetically closer *PhPRR7a*.

The PRR family of Arabidopsis has two conserved domains: PR (Pseudo-receiver Domain) and a CCT (CONSTANS, CONSTANS-like, and TIMING OF CAB EXPRESSION 1 (TOC1/PRR1)) [59] (Figure S3A). PRR5, PRR7, and PRR9 have a repression motif which consists of two regions, L(^E^/_D_)(^L^/_I_)S(^L^/_I_)(^R^/_K_)R and SXXSAF(^S^/_T_)(^R^/_Q_)(^Y^/_F_), located between the PR and CCT domains. They interact with TOPLESS (TPL) and TOPLESS-RELATED PROTEINS (TPRs) [59,60], creating a transcriptional repression complex. This complex represses the transcription of the core genes CIRCADIAN CLOCK ASSOCIATED 1 (*CCA1*) and LATE ELONGATED HYPOCOTYL (*LHY*) [59,61]. We used Arabidopsis as the model, and we compared it with petunia sequences. We found that all the PRR members of *P. axillaris* and *P. inflata* shared the PR domain (Figure S3A). The CCT domain was found in all the coding genes except for PaxiNPRR7b, PinfPRR7a, and PinfPRR7b. The presence of the CCT domain in PaxiNPRR7a and absence from the rest of the gene group in petunia was surprising, thus we analyzed other Solanaceae, member of the Convolvulaceae (*Cuscuta australis* and *Ipomea nil*) and Plantaginaceae (*Antirrhinum majus*). We found that the CCT domain was absent from the PRR7 protein in the Solanaceae analyzed (*Capsicum annuum*, *C. baccatum*, *Nicotiana benthamiana*, *N. sylvestris*, *N. tabacum*, *N. tomentosiformis*, *Petunia axillaris*, *P. inflata*, *Solanum lycopersicum*, *S. melongena*, *S. pennellii*, *S. pimpinellifolium*, and *S. tuberosum*) (Figure S3B). However, the CCT domain was found in the rest of the species analyzed. This indicates an early change in the PRR7 family in Solanaceae with possible implications in clock functioning.

**Figure 2:**
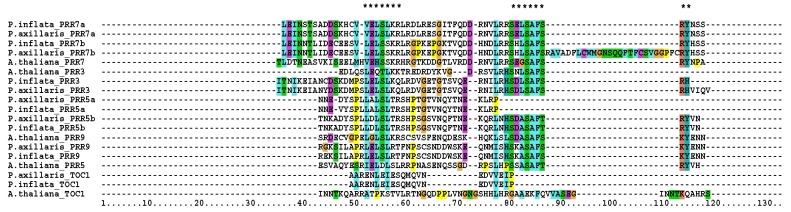
Repression motifs of PSEUDO-RESPONSE REGULATORS (PRRs) local alignment. *Arabidopsis thaliana* (A.thaliana), *Petunia axillaris* (P.axillaris), and *P. inflata* (P.inflata), L(^E^/_D_)(^L^/_I_)S(^L^/_I_)(^R^/_K_)R and SXXSAF(^S^/_T_)(^R^/_Q_)(^Y^/_F_) regions are depicted by asterisks (*).

We also identified the repression motif in *Petunia axillaris* and *P. inflata*, which shared a high homology with Arabidopsis (Figure 2). These motifs are present in PRR5, PRR7, and PRR9 but absent in PRR3 and TOC1 (Figure 2). We found that the paralog PRR5a of *P. axillaris* and *P.inflata* had two major differences. The L(^E^/_D_)(^L^/_I_)S(^L^/_I_)(^R^/_K_)R motif present in the PRR9, 7, and 5 was not canonical as the glutamic/aspartic amino acid of the second position had been replaced by an alanine. Furthermore, both PRR5a had lost the SXXSAF(^S^/_T_)(^R^/_Q_)(^Y^/_F_) motif. We compared the PRR5 proteins of several Solanaceae and Arabidopsis and found that all share the aspartic acid in the second position of the first motif and the complete repression motif absent in PaxiNPRR5a and PinfSPRR5a (Figure S4). This indicates that the D->A amino acid substitution and motif deletion are specific to the Petunia linage.

The SXXSAF(^S^/_T_)(^R^/_Q_)(^Y^/_F_) of PaxiNPRR7b was interrupted by a sequence insertion of 26 amino acids. The sequence of both regions varied among paralogs, as SELSAFSRY was found in the PRR7a lineage and SHLSAFSRY in the PRR7b lineage (Figure 2). Altogether, these changes indicate that PRR duplication occurred before *Petunia* speciation. The absence of the transcriptional repression motif in PRR5a and the interruption of PRR7b in *Petunia inflata* suggest that these paralogs have lost their repression activity.

**Figure 3:**
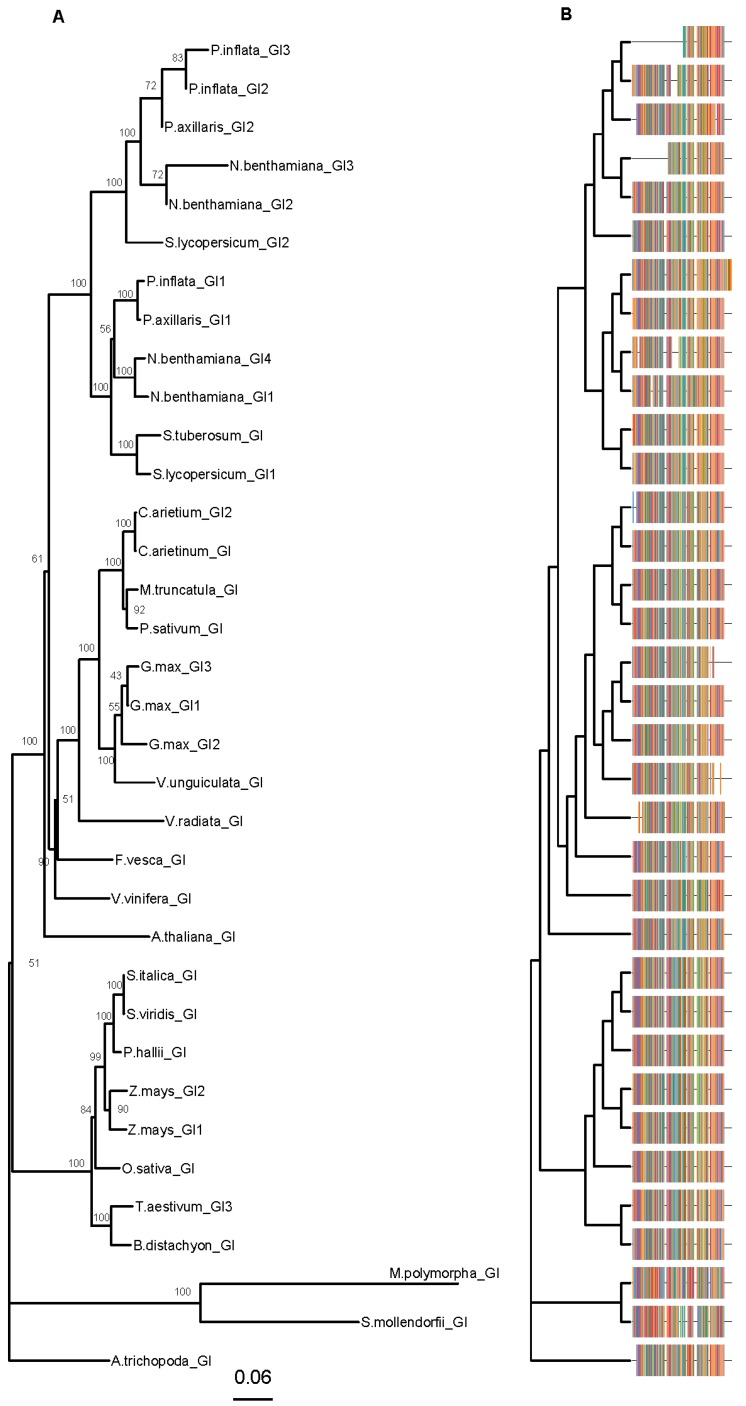
*GIGANTEA* (*GIs*) phylogenetic tree. Amino acid sequences were aligned using CLUSTALX. Phylogenetic analysis was performed using MEGA X and trees were plotted with the library “ggtree” (R version 3.5.1). The phylogenetic trees were built with the neighbor-joining method (NJ) and JTT (Jones, Taylor and Thornton) as model of amino acid substitution. The tree displays the bootstrap percentage (from 500 replicates) next to branches (**A**). The multiple sequence alignment is displayed on the right side (**B**). This tree contains 35 sequences from 25 species. Species abbreviations: A.majus (*Antirrhinum majus*), A.thaliana (*Arabidopsis thaliana*), A.trichopoda (*Amborella trichopoda*), B.distachyon (*Brachypodium distachyon*), C.arietinum (*Cicer arietinum*), F.vesca (*Fragaria vesca*), G.max (*Glycine max*), M.polymorpha (*Marchantia polymorpha*), M.truncatula (*Medicago truncatula*), N.benthamiana (*Nicotiana benthamiana*), O.sativa (*Oryza sativa*), P.axillaris (*Petunia axillaris*), P.hallii (*Panicum hallii*), P.inflata (*Petunia inflata*), P.sativum (*Pisum sativum*), S.italica (*Setaria italica*), S.lycopersicum (*Solanum lycopersicum*), S.moellendorffii (*Selaginella moellendorffii*), S.tuberosum (*Solanum tuberosum*), S.viridis (*Setaria viridis*), T.aestivum (*Triticum aestivum), S.italica (Setaria italica),* V.radiata *(Vigna radiata),* V.unguiculata (*Vigna unguiculata*), V.vinifera (*Vitis vinifera*) and Z.mays (*Zea mays*). Accessions are listed in Table S2.

*GIGANTEA* (*GI*) plays a role in flowering time but is needed to maintain the period and amplitude of clock genes [62,63]. *GI* is a single copy gene in the Arabidopsis genome [62] and it is found in one to three copies in the Solanaceae genomes. *Petunia axillaris* has two copies *PaxiNGI1*, *PaxiNGI2*, whereas *P. inflata* has three copies *PinfS6GI1*, *PinfS6GI2,* and *PinfS6GI3* [11]. Interestingly, the genes *PaxiNGI1* and *PaxiNGI2* are present in the genome of *P. hybrida* Mitchell. We will refer to *GIGANTEA* genes in *P. hybrida* as *PhGI1* and *PhGI2*. The proteins PinfS6GI1 and PaxiNGI1 share an N-terminus conserved with AtGI that was absent in PaxiNGI2 (Figure 3, Figure S5). Furthermore, PaxiNGI2 showed a 41 amino acid insertion that was not conserved in PinfS6GI2 or other *GI* genes. The PinfS6GI3 was much shorter than the other paralogs, a feature conserved in *Nicotiana benthamiana* NbGI3 (Figure 3). The PinfSGI1 had an additional C-terminal fragment of 105 amino acids absent from the rest of the GI genes analyzed (Figure 3, Figure S5). 

We can conclude that the structural evolution of core circadian clock genes and associated genes has occurred at via changes in the number of retained paralogs, gene structure, and coding region.

### 3.2. The Leaf Clock has its Maximum during the Day while the Petal Clock Shifts towards the Night

Several clock models have been described in the last years; one of the most popular plant clocks defines three loops called morning, central, and evening loops. These describe the time of the day when certain genes are preferentially expressed [64]. In Arabidopsis, during the morning *CIRCADIAN CLOCK-ASSOCIATED 1* and *LATE ELONGATED HYPOCOTYL* (*CCA1*/*LHY*) repress *PSEUDO-RESPONSE REGULATOR* members (*PRRs*), which are sequentially expressed (*PRR9*, *PRR7*, *PRR5* and finally *TIMING OF CAB EXPRESSION 1*, *TOC1* or *PRR1*, at dusk) [59]. The *PRR* family represses *CCA1*/*LHY*. *CCA1*/*LHY* also inhibits *GIGANTEA* (*GI*) and the evening complex (EC) expression during the morning [65]. The EC is comprised by *LUX ARRHYTHMO* (*LUX*), *EARLY FLOWERING 4* and *3*, (*ELF4* and *ELF3*). At dusk, TOC1 represses the evening genes *GI*, *ELF4,* and *LUX*. TOC1 interacts with CCA1 HIKING EXPEDITION (CHE) and mediates the repression of *CCA1* [66]. Several activators have been described, including REVEILLE 8 (RVE8) and LIGHT-INDUCIBLE AND CLOCK-REGULATED (LNKs) that positively regulate the transcription of *PRR5* and *TOC1* or the complex LIGHT-REGULATED WD-TEOSINTE BRANCHED 1-CYCLOIDEA-PCF (LWD-TCP), which activates the transcription of *CCA1* [67,68,69]. We established the expression patterns of the different clock genes in leaves and petals. As the genes contained in *P. hybrida* cv Mitchell correspond to *P. axillaris* [11], we further describe them as *Ph* genes. These included the morning loop genes *PhPRR9*, *PhPRR7a*, *PhPRR7b*, *PhPRR5a*, *PhPRR5b,* and *PhPRR3*. The core loop was represented by *PhTOC1* and *PhLHY*. Finally, the evening and clock-associated genes analyzed included *PhGI1*, *PhGI2*, *PhELF4*, *PhCHL* (the *ZEITLUPE* ortholog), and *PhFKF* (*FLAVIN-BINDING KELCH REPEAT FBOX1*). This analysis was performed in petunia that was acclimated to light:dark conditions of 12 h light and 12 h dark (LD) or continuous dark (DD) conditions. 

The non-parametric algorithm RAIN groups gene expression data by their sampling time point e.g., ZT0, ZT3. These groups are assigned to the falling and to the rising part of the oscillation or wave. Then each part, falling and rising, is analyzed by a test against umbrella alternatives [70]. The peak of the umbrella varied, allowing RAIN to test different wave forms. Regardless, if a gene is rhythmically expressed (significant *p*-value) or not, RAIN provides the time point of a peak, if found. We compared two parameters between leaves and petals under a LD cycle: rhythmicity of expression (oscillation) and time point with maximum expression (phase) (Table 1). Concerning rhythmicity, most genes showed a robust oscillation pattern except *PhELF4* and *PhCHL* in leaves, and *PhCHL* in petals, that showed a peak but no significant rhythm (Table 1).

In leaves, most genes had their maximum expression during the light phase, except *PhELF4* that reached its maximum level at ZT12. Petunia clock genes peaked at early morning (*PhLHY*, at ZT0), midday (*PhPRR9* and *PhGI2*, at ZT6), and before dusk (*PhPRR3*, *PhPRR5a*, *PhPRR5b*, *PhPRR7a*, *PhPRR7b*, *PhTOC1*, *PhCHL*, *PhGI1,* and *PhFKF*, at ZT9) (Table 1). In contrast in petals, whereas the clock genes *PhPRR5b*, *PhPRR7a*, *PhPRR7b*, *PhCHL*, *PhELF4,* and *PhLHY* peaked at the same time as observed in leaves, the remaining genes delayed their expression. *PhPRR9* peaked at ZT9 meanwhile *PhPRR5a*, *PhPRR3*, *PhTOC1*, *PhGI1*, *PhGI2,* and *PhFKF* shifted their expression maximum to the dark period. These genes peaked at ZT12 (*PhPRR5a*, *PhGI1*, *PhGI2* and *PhELF4*) and ZT15 (*PhPRR3*, *PhTOC1,* and *PhFKF*) (Table 1). 

### 3.3. Continuous Darkness Shifted the Expression Patterns of Clock Genes

In order to study the entrainment of the petunia circadian clock to the light:dark cycle, petunia plants were transferred from 12 h light:dark (LD) conditions to continuous darkness (DD). We found that *PhPRR7b* and *PhPRR7a* lost their robust oscillation (*p* > 0.05) in leaves and petals, respectively; and as described under a LD cycle, *PhCHL* did not oscillate significantly (*p* > 0.05). In addition, we observed that DD tended to shift the expression patterns of some genes (Table 1,).

**Figure 4:**
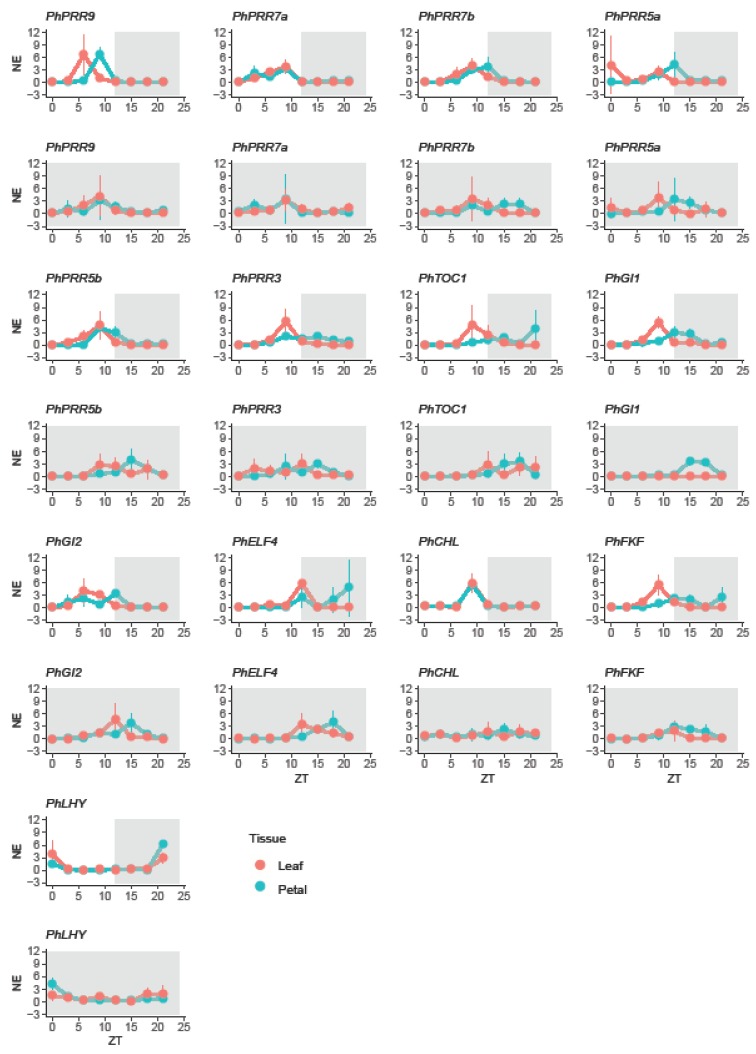

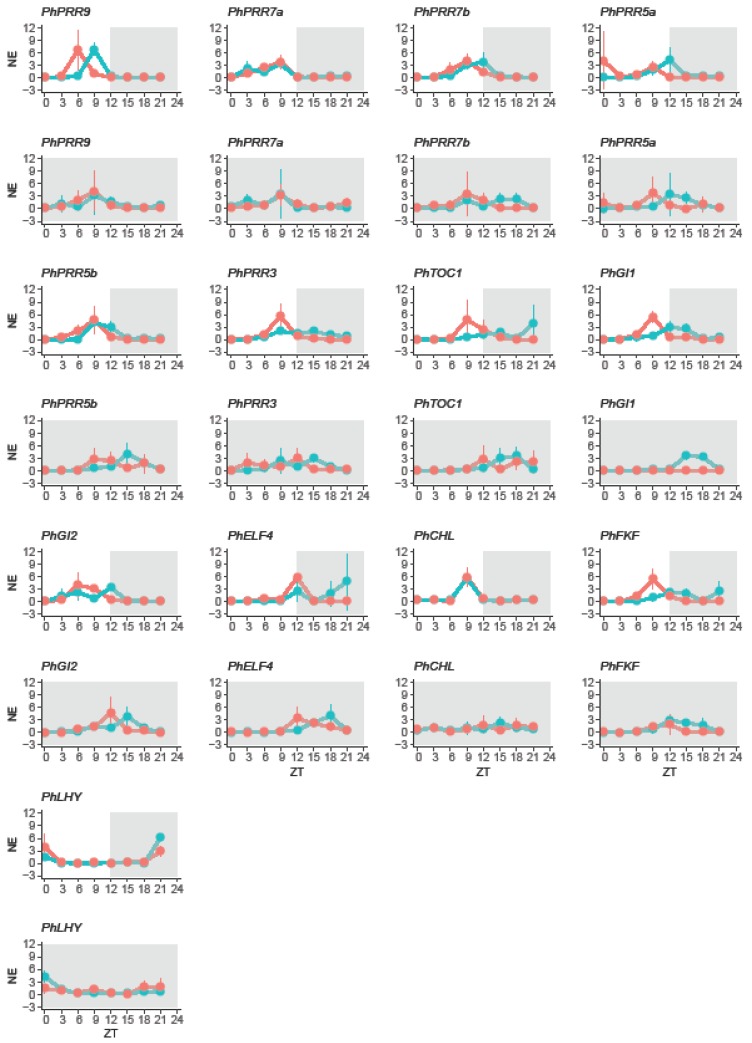
Daily changes in gene expression in petunia. Expression of clock genes in leaves (red) and petals (blue) under a 12 light:dark cycle (LD) and constant darkness (DD). LD cycle is represented by a white and grey area whereas DD cycle is represented by a dark area. Gene expression is represented as mean normalized expression (NE), first every clock gene was normalized to *PhACT* and then, for each time point the expression was divided by the mean expression level. ZT 0 (zeitgeber time) denoting light on, and ZT12, light off. Results represent mean ± SD (*n* = 3).

In leaves, we divided the clock genes into three groups. The first group included *PhPRR5a*, *PhPRR7a*, *PhPRR7b*, *PhFKF,* and *PhLHY*, which maintained their time of maximum expression. The second group included *PhCHL*, advanced by 9 h. Finally, the last group comprised *PhPRR3*, *PhPRR5b*, *PhPRR9*, *PhGI1,* and *PhELF4* delayed by 3 h, *PhGI2* by 6 h, and *PhTOC1* by 15 h (Figure 4, Table 1).

The effect of continuous darkness on petunia petals differed from leaves. Only two genes, *PhPRR3* and *PhLHY* kept their peak or time with maximum expression. *PhPRR7a* and *PhFKF* showed an advanced pattern, peaking 3 h early. Finally, the maximum expression was delayed 3 h for *PhPRR5a*, *PhTOC1*, *PhGI1*, *PhGI2*, 6 h for *PhPRR5b*, *PhPRR9*, *PhCHL,* and *PhELF4* and 9 h *PhPRR7b* (Figure 4, Table 1).

Altogether, *PhLHY* showed a rhythmic oscillation, peaking at early morning regardless of light conditions and tissue. On the other hand, *PhCHL* did not show a rhythmic expression in leaves or petals under light:dark or constant darkness. Besides, we observed that continuous darkness had a different effect on clock genes in leaves and petals, as *PhPRR7b, PhPRR5a,* or *PhPRR3* that showed an organ specific change in phase in response to constant darkness (Table 1). 

### 3.4. Rhythmicity and Photoperiod Sensitivity are Tissue Specific

An important paradigm in the analysis of circadian clock gene expression is the effect of continuous darkness on the genes thought to have a circadian control [71]. We performed an analysis to detect changes in amplitude and phase, in two time series by using Harmonic ANOVA [51]. First, we analyzed differences between tissues by comparing daily gene expression in leaf and petals under light:dark (LD) and under constant darkness (DD). Second, in order to identify the effect on light conditions, we compared LD and DD cycles for leaves and for petals (Table 2). We found that *PhPRR5a, PhPRR7a, PhPRR9,* and *PhLHY* showed a robust expression pattern regardless of the tissue or photoperiod (*p* < 0.05). In contrast, under light:dark conditions, *PhGI1, PhPRR3,* and *PhTOC1* had higher amplitude and were delayed in petals compared to leaves (Table 2). *PhFKF* showed higher amplitude in leaves and was delayed in petals (Table 2). When we compared leaves and petals at LD versus DD, *PhTOC1, PhCHL,* and *PhGI2* in leaves, and *PhPRR7b, PhPRR5b*, *PhCHL*, *PhGI1,* and *PhGI2* in petals, showed a different expression pattern. Interestingly, all these genes were delayed under constant darkness, but differed in their highest amplitude. In leaves, *PhTOC1* displayed the highest amplitude in LD whereas *PhGI2* and *PhCHL*, in DD. In petals, *PhPRR7b* and *PhGI1* amplitude was higher under a light:dark cycle and *PhPRR5b*, *PhGI2,* and *PhCHL* showed their maximum amplitude in DD (Table 2). 

These results indicate that there are two sets of genes with changes in rhythmicity, both in phase and amplitude, in leaves and petals and a group of stable genes comprising *PhPRR5a, PhPRR7a, PhPRR9,* and *PhLHY.* Furthermore, the effect of photoperiod appeared to be organ-specific for those genes that showed significant changes. From all the different genes analyzed, *PhPRR7b* appeared to be specifically affected by DD in petals, indicating a modified control in this organ.

### 3.5. Transcriptional Noise is Gene and Tissue Specific

**Figure 5:**
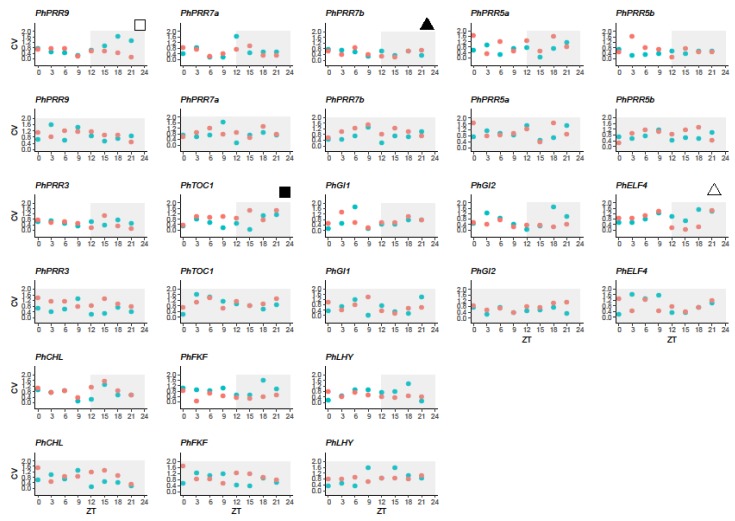
Variability in clock gene expression in petunia leaves (red) and petals (blue) under a light:dark cycle. Coefficient of variation (CV) was calculated for every clock gene and time point for leaves (red) and petals (blue). ZT 0 (zeitgeber time) indicates light on, ZT12 indicates light off. White area represents the light period and grey area, the dark period. The black triangle denotes a gene with low variation in both tissues and the white triangle, a gene with high variation. The white square indicates a gene with high CV in petals whereas the black square denotes a gene with high variation in leaves.

Although gene expression quantities were determined for the same set of mRNA extractions, the degree of significance in terms of gene expression levels was not always as expected based on average expressions. We asked if the variability of gene expression depended on tissue, time, and/or light conditions. When we analyzed the variability under a light:dark cycle (LD) we found that the clock genes *PhPRR7b* and *PhPRR3* showed the lowest variation in leaves and petals whereas *PhELF4* was a highly variable gene in both tissues. The stability of remaining genes varied among tissues, such as *PhPRR9* or *PhTOC1*, showing lower transcriptional noise in leaves than petals. In addition, we observed that the lowest and the highest variability for all analyzed genes varied along the day, suggesting that transcriptional noise may be time-dependent (Figure 5, Figure S6).

When plants were transferred to constant darkness (DD), we found that the variability of clock genes tended to increase, except for *PhLHY* in leaves and *PhGI2* in petals that were the most stable genes. Interestingly, the increase in CV was higher in leaves (Figure 5, Figure S6). The genes *PhPRR7a*, *PhPRR7b*, *PhPRR3,* and *PhFKF* showed the highest variability under constant darkness. In petals, the transcriptional noise of *PhPRR5a* and *PhTOC1* increased under a DD cycle (Figure 5). As previously mentioned, under a light:dark cycle, the coefficient of variation changed depending on the time point. The highest and lowest noise for leaves coincided with early and late day respectively, while in petals transcriptional noise was low in the subjective night and higher noise was found at subjective time ZT9. 

We used a scale test (HarmScaleTest) implemented in the DODR package [51] in order to detect significant changes in noise when petunia was transferred to constant darkness (Table 3). The CV was in general higher under constant darkness, suggesting that the transcriptional noise tended to increase in DD. The exceptions were *PhGI1* and *PhELF4* in leaves and *PhCHL*, *PhFKF, PhGI1,* and *PhGI2* in petals, displaying slightly higher CV under a LD cycle. We confirmed that the effect was tissue dependent, as changes in CV were significant (*p* < 0.05) in leaves for *PhPRR7b*, *PhFKF,* and *PhGI1* and in petals, for *PhPRR7a*, *PhPRR3,* and *PhELF4* (Table 3). The only gene affected in leaves and petals was *PhCHL*, but it also displayed opposite trends of increased CV (Table 3). This indicates that an endogenous component governs transcriptional noise of the clock genes, which also differs in leaves and petals.

## 4. Discussion

### 4.1. The Petunia Clock Genes Show Structural Evolutionary Changes

The evolution of the plant circadian clock is considered an important driver of adaptation in a variety of plants including tomato, *Opuntia ficus-indica*, or barley [40,72,73,74]. The plant clock is an important coordinator of primary and secondary metabolism in plants. It defines the timing of floral scent emission and floral blend in a variety of plants including *Antirrhinum majus, Nicotiana attenuatta*, or *Petunia* [35,36,37,38]. The plant circadian clock appears to have a specific transcriptional structure in different tissues such as leaves, pods, seeds, or roots [13,14,75,76]. As the transcriptional structure of the clock in petals is currently unknown, we used *Petunia hybrida* to perform a detailed analysis. We characterized the structural changes in *PhPRR5a*, *PhPRR5b, PhPRR7a*, *PhPRR7b*, *PhGI1,* and *PhGI2* and the transcriptional structure of the petunia circadian clock and associated genes in petals and leaves, using standard growth and continuous darkness.

The complete genome paleohexaploidization of petunia, found in the Solanaceae group [11] is reflected in the retaining of several clock genes as duplications that are found as single copy genes in Arabidopsis and other species. These include *PhPRR5a*, *PhPRR5b, PhPRR7a*, *PhPRR7b*, *PhGI1,* and *PhGI2.* Other genes that are found as single copy include *PhLHY, PhPRR9, PhPRR3, PhTOC1, PhFKF,* and *PhCHL* the ortholog of *ZTL*. Interestingly, genes found as single copy in petunia such as *PhTOC1*, *PhPRR9,* and *PhPRR3* are found as single copy in most Solanaceae except for *Nicotiana benthamiana* that appears to have two copies of each gene (Figure 1). Two of the petunia paralogs of *AtPRR7*, *PhPRR7a*, *PinfS6PRR7a* and the ones corresponding to *AtPRR5 PhPRR5a,* and *PinfS6PRR5a* cluster between Arabidopsis and the rest of the Solanaceae genes. In contrast the single copy genes *TOC1*, *PRR3,* and *PRR9* are found as a subclade for all the Solanaceae together including *Petunia*. This indicates that there has been a loss of *PRR5* and *PRR7* paralogs in the Solanaceae that have a single copy gene, while *Petunia* has retained the older copy closer to the Arabidopsis, *Vitis vinifera,* and *Amborella trichopoda* genes. The additional changes observed in the number of exons indicate a specific evolution of one paralog. Indeed, *AtPRR5* has six exons whereas *AtPRR7* presents nine exons (NP_568446.2 and AT5G02810, consulted in NCIB and TAIR database) while *PhPRR5a* and *PhPRR7b* present seven exons whereas *PhPRR5b* and *PhPRR7a* have eight exons, indicating possible sub or neofunctionalization of these paralogs (see below).

We found two domains, PR and CCT in all analyzed TOC1, PRR3, PRR5, and PRR9 sequences. In contrast, the CCT domain was absent in most PRR7 paralogs in *Capsicum* spp., *Petunia* spp., *Solanum* spp. and *Nicotiana* spp. Interestingly, we only found the CCT domain in PhPRR7a, which shared more similarities in the amino acids sequence with AtPRR7. The lack of CCT domains in Solanaceae but not in the related Convolvulaceae family suggests that this event occurred in the early history of Solanaceae. In addition, this alteration, which has been described in PRR orthologs in crops such as rice and soybean, can modify growth and flowering time [77,78]. This may result in a specific clock in the Solanaceae family.

The current hypothesis about the transcriptional structure of the plant circadian clock includes a three component repressilator structure [64]. The PRR9, 7, and 5 genes function as transcriptional repressors in Arabidopsis [59]. The interaction between PRR9, 7, and 5 with TOPLESS occurs via two motifs. We found that the repression motifs were lost in the PhPRR5a and PinfS6PRR5a but was present in the rest of the paralogs of the PRR9, 5, and 3 genes. In addition, in *P. axillaris* PRR7b repression motif was interrupted by a sequence of 26 amino acids. This may result in the loss of gene repression activity of PRR5a and PRR7b [79]. Our results thus indicate that one of the repressilator loops in Petunia is maintained by one of the paralogs, while the second one PRR5a and PRR7b missing the repression domain may have undergone neofunctionalization.

The gene *GI* appeared in land plants and is absent in mosses or picoalgae [80]. In the Solanaceae we found two to three copies, and in *Petunia hybrida*, there are significant differences in the coding region between *PhGI1* and *PhGI2* suggesting a diversification of functions. Furthermore, the amino acid differences between *P. axillaris* and *P. inflata* indicate species specific changes in this master regulator that may be related to the differing environmental niches where both species grow.

We used the predicted protein sequences to infer the domain structure of GIGANTEA. Although a previous study describes that *GI* encodes a protein with six transmembrane domains [63], the biochemical functions of GI are not understood. In yeast, two hybrid experiments performed with the Arabidopsis GI protein show that the N-terminal domain interacts with FKF1 [81], while the complete protein shows interactions with the CYCLING DOF FACTOR6 and DELLA protein [82,83]. As the differences in protein structure found between PhGI1 and PhGI2 do not match well known domains we cannot understand their functional differences. Nevertheless, the PinfS6GI3 does lack the N terminus required for interactions with FKF1 and ZTL in Arabidopsis.

### 4.2. Daily Expression of Petunia Clock Genes is Tissue Specific

The current transcriptional model of the plant circadian clock is largely based on the expression of genes in the Arabidopsis hypocotyls and leaves [84]. It includes the morning, midday or core, and the evening loops. During the morning, the genes *CCA1* and *LHY* repress the evening genes *GI* and *TOC1* and activate *PRR9* and *PRR7*. At the same time, *TOC1* acts repressing *GI* and *PRR9* but activating *CCA1/LHY*. On the other hand, GI stabilizes ZTL that is a *TOC1* repressor [85].

Previous studies have revealed that the circadian clock is tissue-specific [14,75,76]. Differential expression of clock genes has been reported in several tissues including seeds, roots, leaves, stems, and flowers at several developmental stages in different plant species such as bamboo [86], radish [87], or daisy [88]. 

Changes in gene expression of timing, quantity, and rhythm may hint at possible subfunctionalization or neofunctionalization of duplicated clock genes. We found that gene expression patterns of *PhPRR5* and *PhGI* paralogs differed in petals and leaves respectively, under a light:dark cycle. *PhPRR5b* and *PhGI2* displayed an advanced phase, peaking before *PhPRR5a* and *PhGI1*. Under DD, *PhPRR5b* was delayed 3 h in leaves. In petals, *PhPRR7a* advanced 6 h while PhPRR7b delayed 9 h. Given the differences in protein structure observed between PhPRR5a, PhPRR5b, PhPRR7a, and PhPRR7b, and the drastic changes in expression pattern we can conclude that these paralogs have undergone important changes in biological function. As changes are tissue specific, it appears that the coordination of the clock in petals differs from the canonical expression in leaves.

### 4.3. Leaves and Petals have Different Clock Coordination

The current hypothesis is that several independent inputs such as light, temperature, or sugar act as signals helping the plant clock to remain stable. One of the signals considered differing between tissues is light [89]. In the present work we explored oscillations in gene expression in leaves and petals that do not differ in their exposure to light or temperature. We used the RAIN algorithm, a non-parametric method which also provided measures of phase [50]. We also performed an HANOVA test comparing tissues and light conditions. The core clock genes *LHY* and *TOC1* are found in basal picoeukaryotes, mosses, *Marchantia polymorpha*, and all higher plants [5,80,90]. We found that *PhLHY* and *PhPRR9* did not show any statistical differences regardless the tissue or light cycle. In contrast, *PhTOC1* expression pattern differed between leaves and petals in LD, i.e., standard growing conditions. This indicates a basal change in the clock coordination between both tissues. This scenario may be further supported by the significant changes found for *PhFKF*, *PhPRR3*, *PhGI1,* and *PhGI2* between tissues. Finally, *PhGI1*, a gene found only in flowering plants showed significant changes between tissues and photoperiods indicating that it may play a role in the coordination between development and environmental signals. An initial hypothesis proposed different clock structures in leaves and roots of Arabidopsis based on the light reception by the organs [14]. However work performed in cowpea shows that aerial organs exposed to light such as leaves, pods, or developing seeds show different transcriptional clock structures [76]. Our results indeed indicate a differential coordination of the leaf and petal clock that may be the result of the establishment of the petal identity by the floral organ identity genes.

### 4.4. Photoperiod Sensitivity is Organ-Specific

The effect of day length on biological clocks has been widely studied. For example, floral transition is controlled by *CONSTANS* (*CO*) and *FLOWERING LOCUS T* (*FT*) genes which are regulated by the circadian clock, including *ELF3*, *ELF4*, *GI, LHY, PRRs,* and *ZTL* genes [91,92,93]. These genes are capable to integrate environmental cues, mainly day length, but also temperature. Clock genes are therefore sensitive to ambient changes resulting in an adaptive advantage [85]. The present study revealed that a constant dark regime induced phase-shift even in the first 24 h. Most analyzed genes tended to delay their maximum expression, especially in leaves. Only *PhLHY* did not change its expression and displayed a robust oscillation in leaves and petals. Other genes, *PhPRR7a* and *PhELF4* (in leaves), did not retain their rhythmicity, suggesting that the integration of environmental cues and phototransduction varies depending on the tissue. This is consistent with previous studies, that have reported the effect of light on organ-specific circadian clocks and photoperiodic sensitivity [14,94].

Constant dark also had an effect on oscillations, which in general tended to decrease in most analyzed genes in leaves and petals. Similar results have been reported in other plant species: *LHY/CCA1*, *ELF4*, *GI,* and *TOC1* gene expression dampens under constant light or constant dark conditions in Arabidopsis [35,63,95,96]. Loss of circadian rhythmicity could be key and be involved in responses to environmental changes, such as seasonal dormancy during winter in Japanese cedar or chestnut [97,98].

### 4.5. Transcriptional Noise is Tissue-Specific and Depends on Photoperiod

One of the main features of the transcriptional structure of circadian clocks is the capacity to integrate noisy environmental signals and internal transcriptional variation [99]. The robustness of circadian oscillation is related to the number of mRNA molecules, interactions, and complex formation, and it is stabilized by the entrainment to the light:dark cycle [100]. 

In the present work we used a set of biological samples to analyze 13 genes under two light conditions and two tissues. We found that for the same set of samples some genes showed very high noise at a given time of the day, while others were very stable (Figure 5). We also found that molecular noise differed in leaves and petals and it was influenced by the time of the day. While in leaves highest stability appeared at the beginning of the subjective day, petals displayed the lowest stability. This was also noticeable when plants were transferred to continuous darkness. Interestingly, the time point with the highest transcriptional noise shifted both in leaves and petals. The lowest stability advanced in petals, and delayed in leaves. Furthermore, the increased transcriptional robustness early in the day in leaves, and in the late day-early night in petals, coincide with the major functional changes in both tissues, initiation of photosynthesis, and scent emission. As noise increases thereafter in both tissues, it could be that funneling transcriptional noise into robustness at certain times of the day may have biological implications to achieve consistent outputs. However, the molecular function, if any, is not understood as this is the first report of this phenomenon.

Taken together the differential transcriptional structure and response to light, we conclude that the circadian clock in leaves and petals show substantial differences, that may reflect the underlying function in controlling photosynthesis and secondary metabolism in both tissues. The functional differences between leaves and petals may rely in part on a circadian clock reprogramming during flower development.

## Figures and Tables

**Table 1:** Rhythmic analysis of transcriptional data. Detection of rhythmicity in petunia clock genes in leaves and petals under a light:dark cycle (LD) and constant darkness (DD). This analysis was performed with a non-parametric method implemented in the Bioconductor package “RAIN”. Significant *p*-values (*p* < 0.05) indicate a robust oscillation. Phase denotes the time point with the maximum gene expression or peak.

Gene	LD				DD				
	Leaf		Petal		Leaf			Petal	
	*p*-value	Phase	*p*-value	Phase	*p*-value	Phase	*p*-value	*p*-value	Phase
*PhPRR9*	0.000	6	0.000	9	0.001	9	0.007	12	0.000
*PhPRR7a*	0.000	9	0.037	9	0.007	9	0.094	3	0.000
*PhPRR7b*	0.000	9	0.000	9	0.330	9	0.006	18	0.000
*PhPRR5a*	0.008	9	0.000	12	0.001	9	0.000	15	0.008
*PhPRR5b*	0.000	9	0.000	9	0.016	12	0.000	15	0.000
*PhPRR3*	0.000	9	0.000	15	0.143	12	0.000	15	0.000
*PhTOC1*	0.000	9	0.001	15	0.017	21	0.000	18	0.000
*PhGI1*	0.000	9	0.007	12	0.000	12	0.000	18	0.000
*PhGI2*	0.000	6	0.000	12	0.000	12	0.000	15	0.000
*PhELF4*	0.074	12	0.011	12	0.000	15	0.000	18	0.074
*PhCHL*	0.251	9	0.059	9	0.380	0	0.075	15	0.251
*PhFKF*	0.000	9	0.004	15	0.001	9	0.000	12	0.000
*PhLHY*	0.000	0	0.030	0	0.005	0	0.000	0	0.000

**Table 2:** Analysis of differential gene expression in petunia leaves and petals under two light conditions: light:dark (LD) and constant darkness (DD). We compared the daily gene expression of both tissues (petal vs. leaf), under light:dark and constant darkness. We also compared every single tissue under two light conditions (LD vs. DD). This analysis uses Harmonic ANOVA (HANOVA) to test differences in amplitude and phase. A *p* value < 0.05 indicated that the amplitude and/or phase were significantly different between tissues (petal vs. leaf) or between light conditions (LD vs. DD). Amp displays the tissue with the higher amplitude and Phase symbols + and – denotes delayed and advanced, respectively, per tissue or experimental condition.

	Petal vs. Leaf						LD vs. DD					
Gene	LD	Amp	Phase	DD	Amp	Phase	Leaf	Amp	Phase	Petal	Amp	Phase
*PhPRR9*	0.364	Leaf	+3 h petal	0.626	Leaf	+3 h petal	0.588	DD	+3 h DD	0.459	DD	+3 h DD
*PhPRR7a*	0.588	Leaf	No changes	0.700	Leaf	–6 h petal	0.161	LD	No changes	0.988	LD	–6 h DD
*PhPRR7b*	0.196	Leaf	No changes	0.094	Leaf	+9 h petal	0.876	LD	No changes	0.035	LD	+9 h DD
*PhPRR5a*	0.061	Petal	+3 h petal	0.175	Petal	+6 h petal	0.464	LD	No changes	0.616	LD	+3 h DD
*PhPRR5b*	0.223	Leaf	No changes	0.169	Petal	+3 h petal	0.064	LD	+3 h DD	0.004	DD	+6 h DD
*PhPRR3*	0.014	Petal	+6 h petal	0.151	Petal	+3 h petal	0.330	LD	+12 h DD	0.872	LD	No changes
*PhTOC1*	0.012	Petal	+6 h petal	0.638	Petal	–3 h petal	0.033	LD	+3 h DD	0.399	LD	+3 h DD
*PhGI1*	0.019	Petal	+3 h petal	0.109	Petal	+6 h petal	0.080	LD	+3 h DD	0.009	LD	+6 h DD
*PhGI2*	0.291	Petal	+6 h petal	0.298	Petal	+3 h petal	0.041	DD	+6 h DD	0.012	DD	+3 h DD
*PhELF4*	0.049	Leaf	No changes	0.131	Petal	+3 h petal	0.135	DD	+3 h DD	0.739	DD	+6 h DD
*PhCHL*	0.981	Petal	No changes	0.803	Leaf	+15 h petal	0.037	DD	+3 h DD	0.042	DD	+6 h DD
*PhFKF*	0.003	Leaf	+6 h petal	0.437	Petal	+3 h petal	0.479	LD	No changes	0.318	DD	–3 h DD
*PhLHY*	0.675	Leaf	No changes	0.205	Petal	No changes	0.254	LD	No changes	0.137	DD	No changes

**Table 3:** Detection of changes in noise in petunia clock genes in two tissues, leaves and petals, comparing light:dark (LD) and constant darkness (DD) cycles. This analysis was performed by using the function HarmScaleTest provided by the R package DODR. A *p* value < 0.05 indicates significant changes between LD and DD, the phase with higher variability is specified in Noise columns.

Gene	Leaf, LD vs. DD	Noise	Petal, LD vs. DD	Noise
*PhPRR9*	0.341	DD	0.508	DD
*PhPRR7a*	0.228	DD	0.002	DD
*PhPRR7b*	0.014	DD	0.958	DD
*PhPRR5a*	0.137	DD	0.312	DD
*PhPRR5b*	0.839	DD	0.805	DD
*PhPRR3*	0.385	DD	0.004	DD
*PhTOC1*	0.575	DD	0.137	DD
*PhGI1*	0.000	LD	0.580	LD
*PhGI2*	0.301	DD	0.860	LD
*PhELF4*	0.082	LD	0.005	DD
*PhCHL*	0.016	DD	0.007	LD
*PhFKF*	0.013	DD	0.124	LD
*PhLHY*	0.140	DD	0.084	DD

## Supplementary Materials

The following are available online at https://www.mdpi.com/2073-4425/10/11/860/s1, Figure S1: Metl or dissociation curve analysis of petunia genes, Figure S2: Exon-intron structure of *Petunia axillaris* (PaxiN) *PRR5* and *PRR7* genes, Figure S3: Domain structure of PSEUDO-RESPONSE REGULATORS (PRRs) , Figure S4: Local alignment of repression motifs of PRR5, Figure S5: Local alignment of GIGANTEA (GIs) proteins, Figure S6: Normalized expression (NE) for the biological replicates, Table S1: PSEUDO-RESPONSE REGULATORS (PRRs) protein accessions used in the phylogenetic reconstruction and for the annotation of protein sequences, Table S2: GIGANTEA (GI) protein accessions used in the phylogenetic reconstruction, Table S3: Primers used for qPCR,
