# Supplementary material for: Transcriptional Structure of Petunia Clock in Leaves and Petals
Source: Genes (Basel). 2019 Oct 30;10(11):860. doi: 10.3390/genes10110860 (PMC6895785; doi:10.3390/genes10110860)
Supplement: Supplementary file 1 [file genes-10-00860-s001.pdf]

Transcriptional structure of petunia clock in leaves and petals.

Marta I. Terry, Marta Carrera-Alesina, Julia Weiss, Marcos Egea-Cortines

Supplementary Information

Figure S1. Melt or dissociation curve analysis of petunia genes.

Figure S2. Exon-intron structure of *Petunia axillaris* (PaxiN) *PRR5* and *PRR7* genes.

Figure S3. Domain structure of PSEUDO-RESPONSE REGULATORs (PRRs) proteins.

Figure S4. Local alignment of repression motifs of PRR5 proteins.

Figure S5. Local alignment of GIGANTEA (GIs) proteins.

Figure S6. Normalized expression (NE) for the biological replicates.

Table S1. PSEUDO-RESPONSE REGULATORs (PRRs) protein accessions used in the phylogenetic reconstruction and for the annotation of protein sequences.

Table S2. GIGANTEA (GI) protein accessions used in the phylogenetic reconstruction.

Table S3. Primers used for qPCR.

Fig. S1. Melt or dissociation curve analysis of petunia genes.

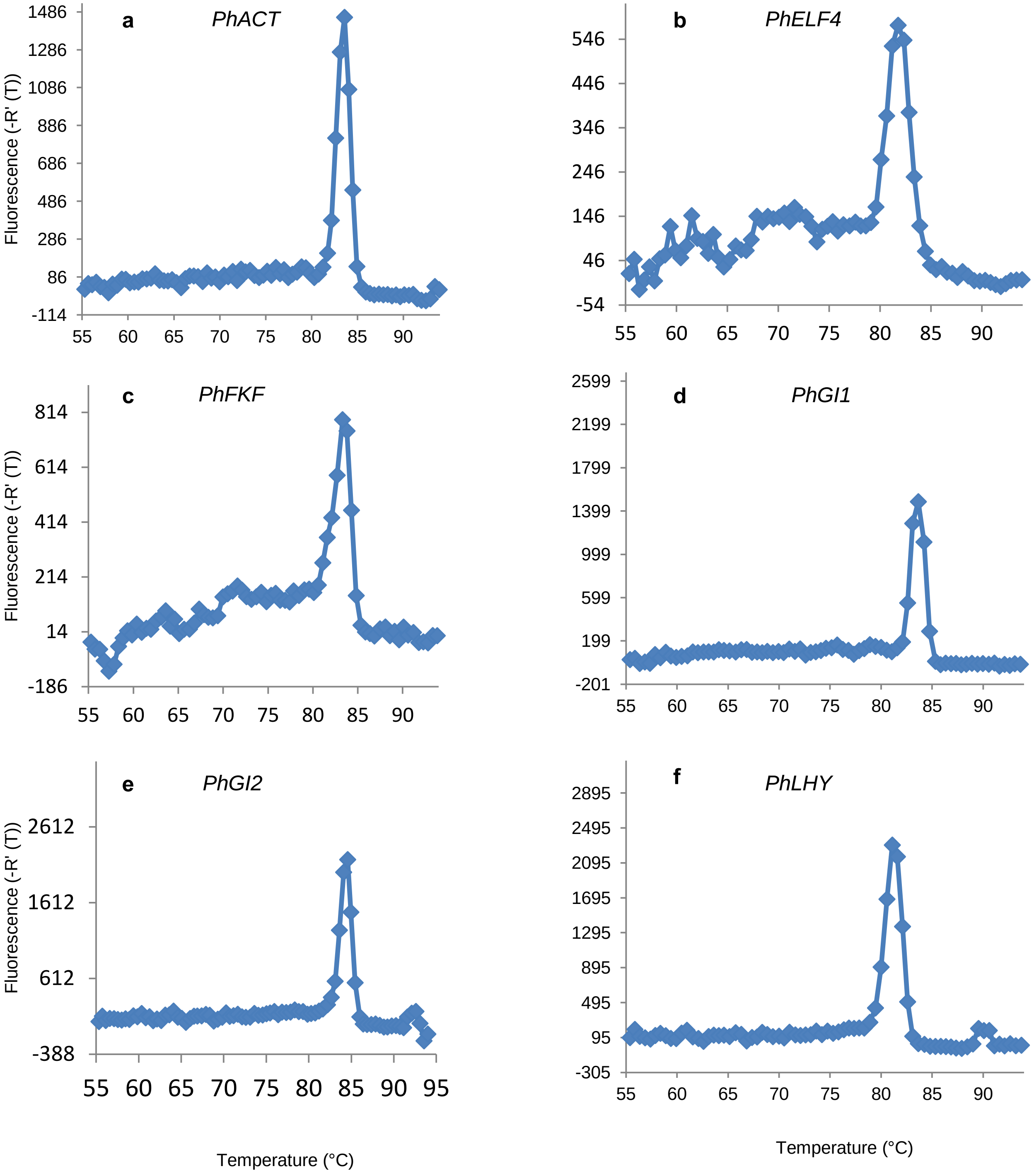

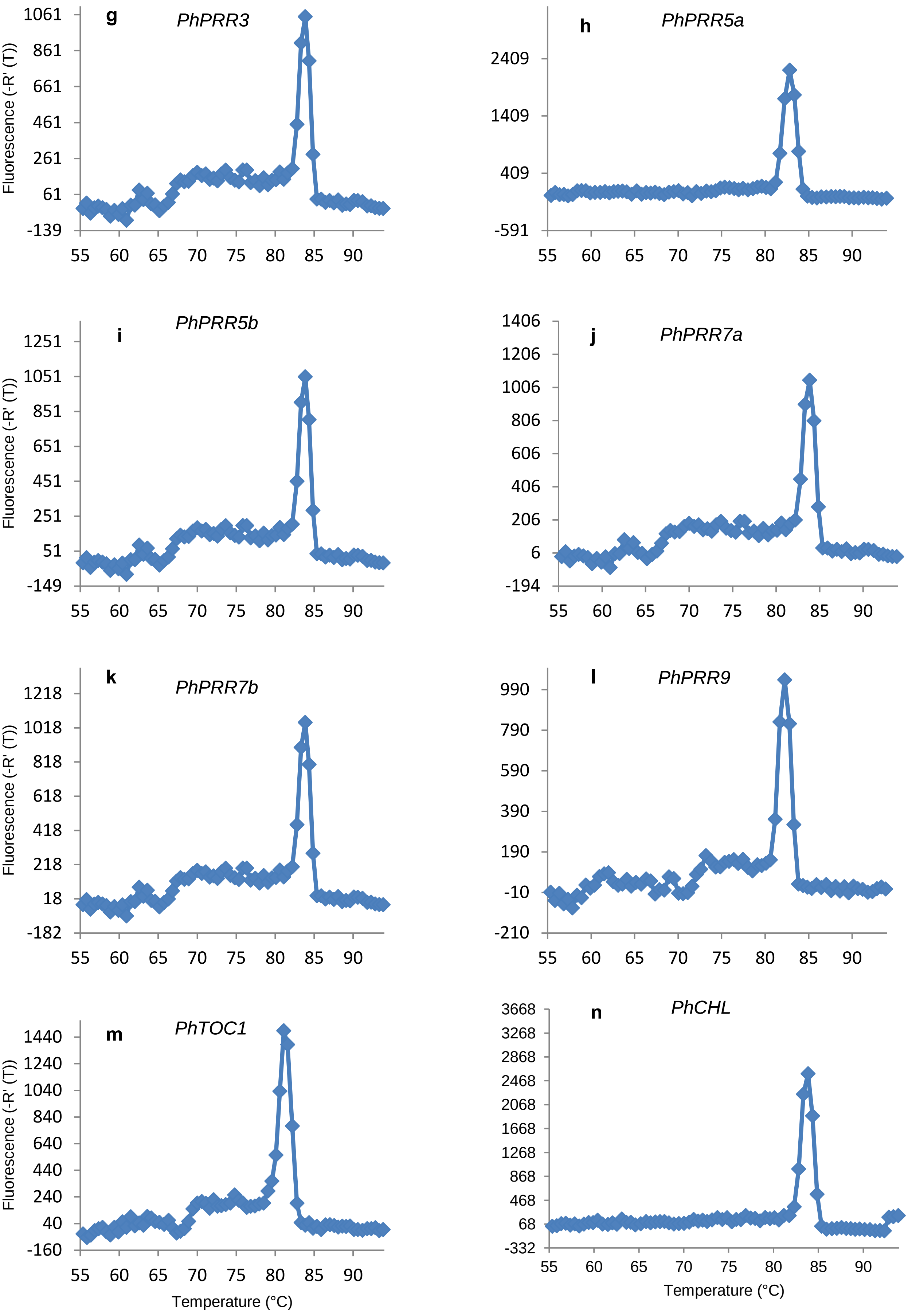
g
PhPRR3
h
PhPRR5a
i
PhPRR5b
j
PhPRR7a
k
PhPRR7b
l
PhPRR9
m
PhTOC1
n
PhCHL
Fluorescence (-R' (T))
Temperature (°C)
1061
861
661
461
261
61
-139
2409
1409
409
-591
1251
1051
851
651
451
251
51
-149
1406
1206
1006
806
606
406
206
6
-194
1218
1018
818
618
418
218
18
-182
990
790
590
390
190
-10
-210
1440
1240
1040
840
640
440
240
40
-160
3668
3268
2868
2468
2068
1668
1268
868
468
68
-332
55
60
65
70
75
80
85
90

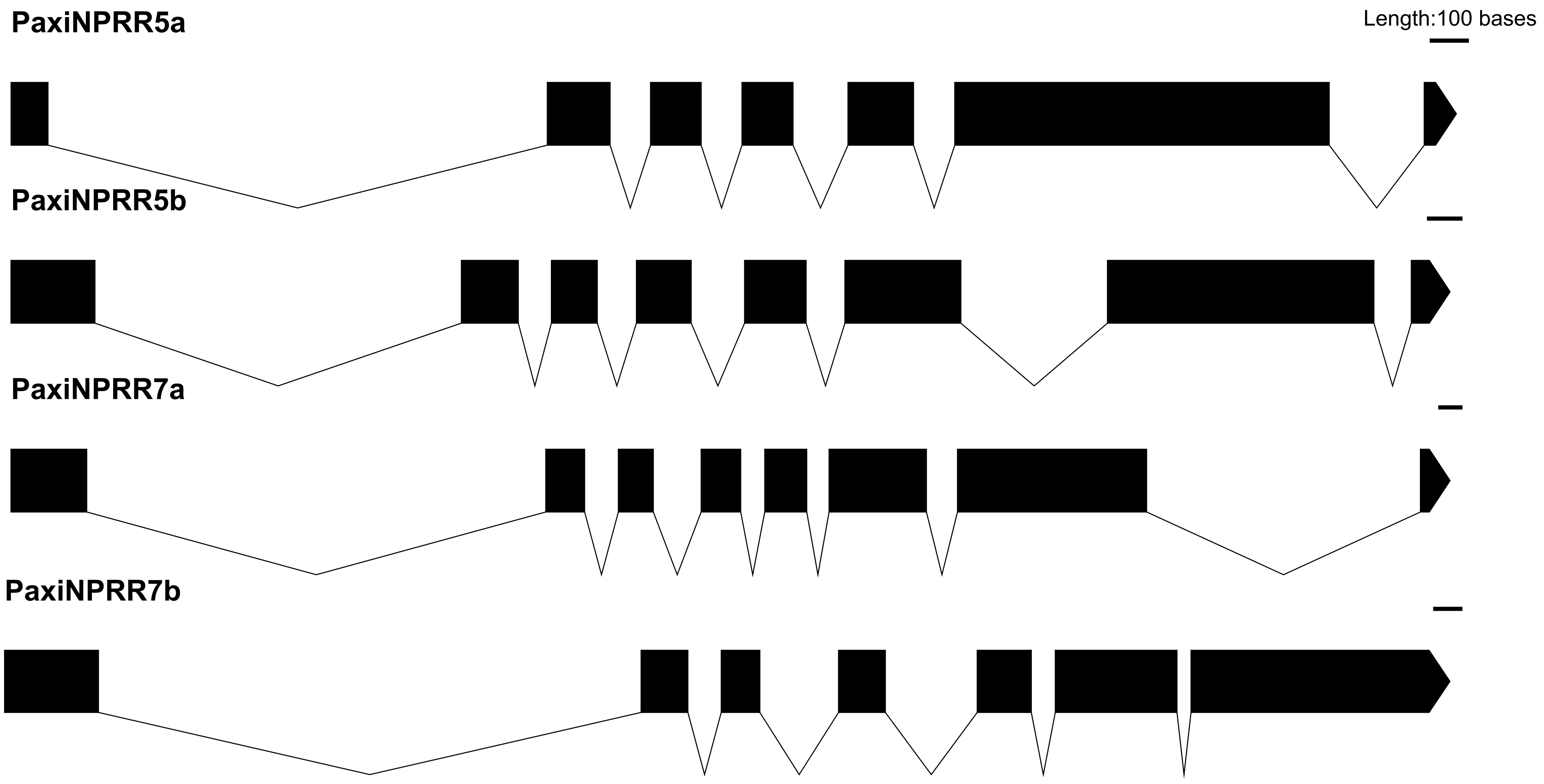

Fig. S2. Exon-intron structure of Petunia axillaris (PaxiN) PRR5 and PRR7 genes. The exon-intron boundary was calculated using the predicted mRNA molecules from petunia and aligning them to the corresponding genomic sequences.

Fig. S3. (a) Domain structure of PRRs proteins in *Arabidopsis thaliana* (At), *Petunia axillaris* (Pa) and *P. inflata* (Pi). (b) Domain structure of PRR7s proteins in *Arabidopsis thaliana* (At), *Capsicum annuum* (Ca), *C.baccatum* (Cb), *Nicotiana benthamiana* (Nb), *N.sylvestris* (Ns), *N.tabacum* (Nta), *N.tomentosiformis* (Nto), *Petunia axillaris* (Pa), *P. inflata* (Pi), *Solanum lycopersicum* (sl), *S.melongena* (Sm), *S.pennellii* (Spe), *S.pimpinellifolium* (Spi), *S.tuberosum* (St), *Cuscuta australis* (Cuas), *Ipomea nil* (In) and *Antirrhinum majus* (Am). Accessions are listed in Supplementary Table S1.

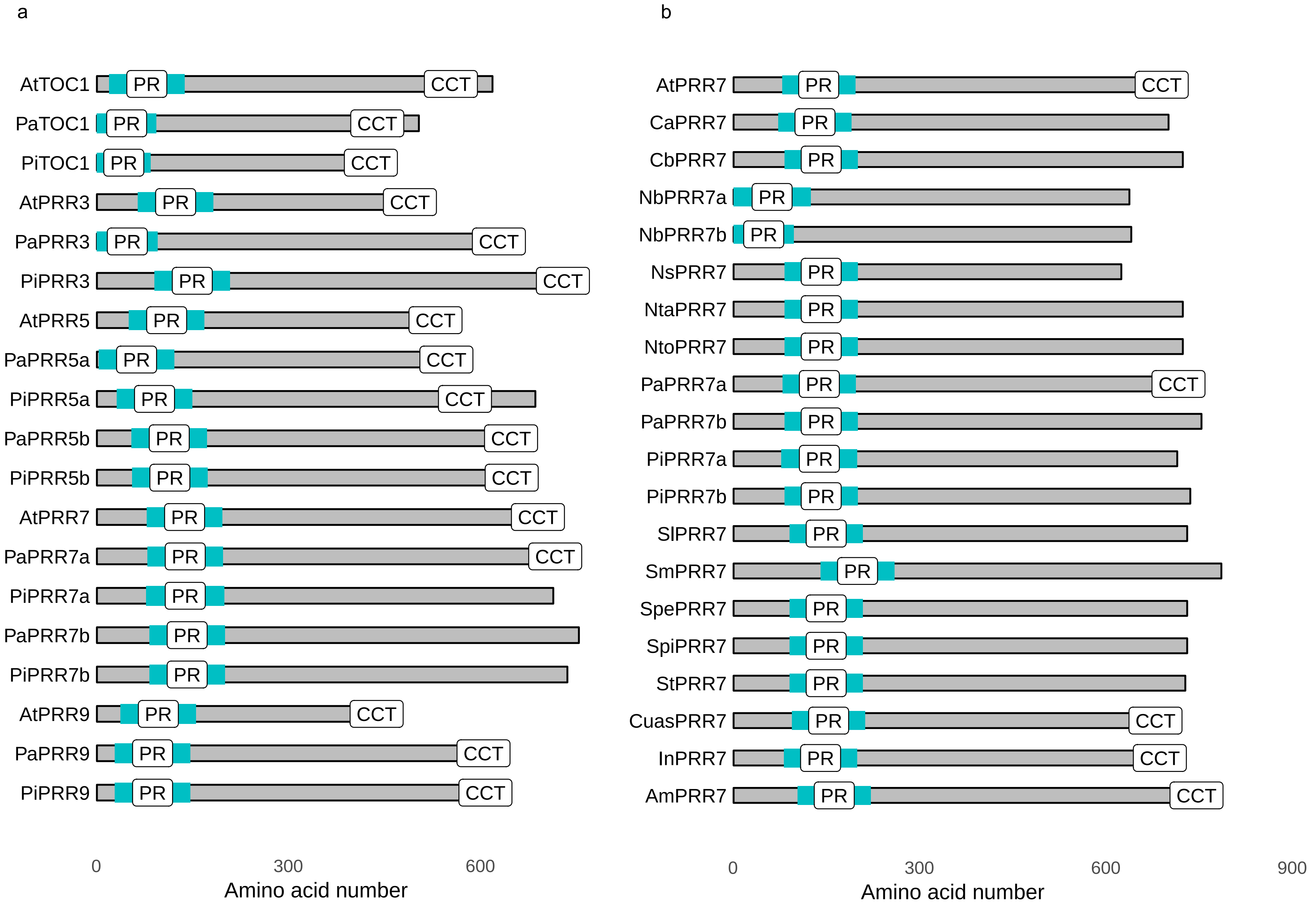
a
AtTOC1
PaTOC1
PiTOC1
AtPRR3
PaPRR3
PiPRR3
AtPRR5
PaPRR5a
PiPRR5a
PaPRR5b
PiPRR5b
AtPRR7
PaPRR7a
PiPRR7a
PaPRR7b
PiPRR7b
AtPRR9
PaPRR9
PiPRR9
PR
CCT
0
300
600
Amino acid number
b
AtPRR7
CaPRR7
CbPRR7
NbPRR7a
NbPRR7b
NsPRR7
NtaPRR7
NtoPRR7
PaPRR7a
PaPRR7b
PiPRR7a
PiPRR7b
SlPRR7
SmPRR7
SpePRR7
SpiPRR7
StPRR7
CuasPRR7
InPRR7
AmPRR7
PR
CCT
0
300
600
900
Amino acid number

Fig.S4. Local alignment of repression motifs of PRR5 proteins. *Arabidopsis thaliana* (A.thaliana), *Capsicum annuum* (C.annuum), *Nicotiana benthamiana* (N.benthamiana), *N. tomentosiformis* (N.tomentosiformis), *N. sylvestris* (N.sylvestris), *Petunia axillaris* (*P.axillaris)*, *P. inflata* (P.inflata), *Solanum lycopersicum* (S.lycopersicum) and *S. tuberosum* (S.tuberosum).

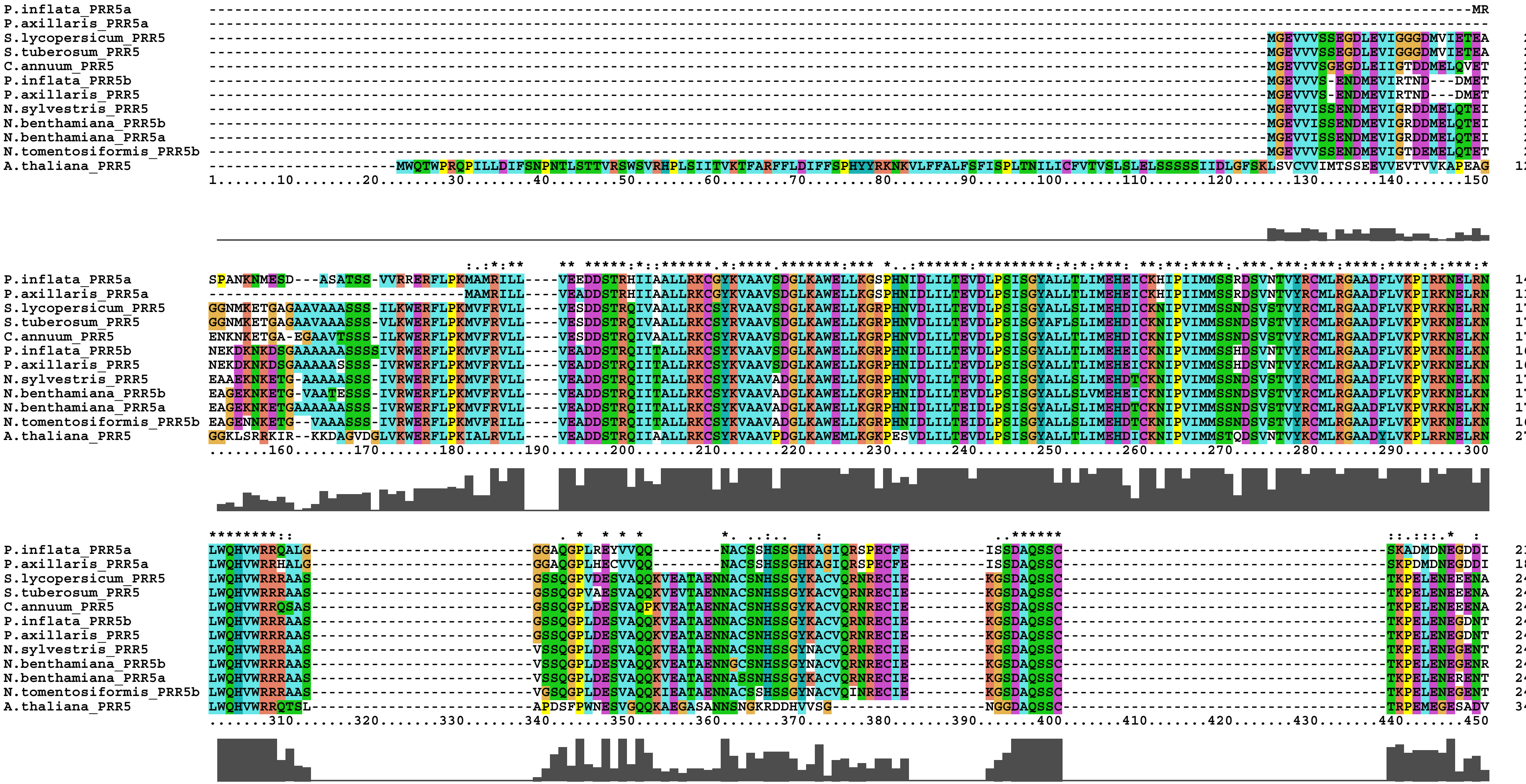

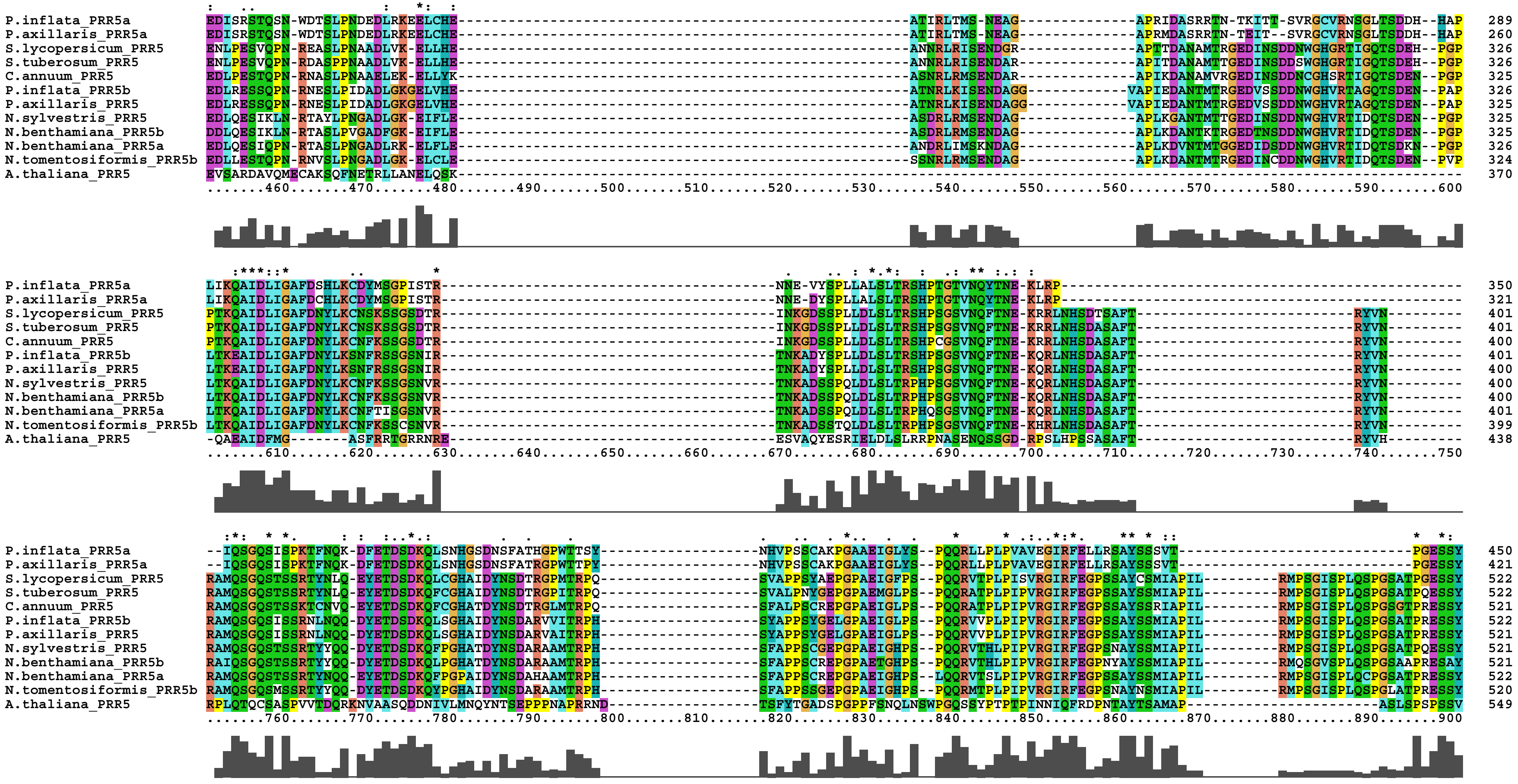
P.inflata_PRR5a
P.axillaris_PRR5a
S.lycopersicum_PRR5
S.tuberosum_PRR5
C.annuum_PRR5
P.inflata_PRR5b
P.axillaris_PRR5
N.sylvestris_PRR5
N.benthamiana_PRR5b
N.benthamiana_PRR5a
N.tomentosiformis_PRR5b
A.thaliana_PRR5

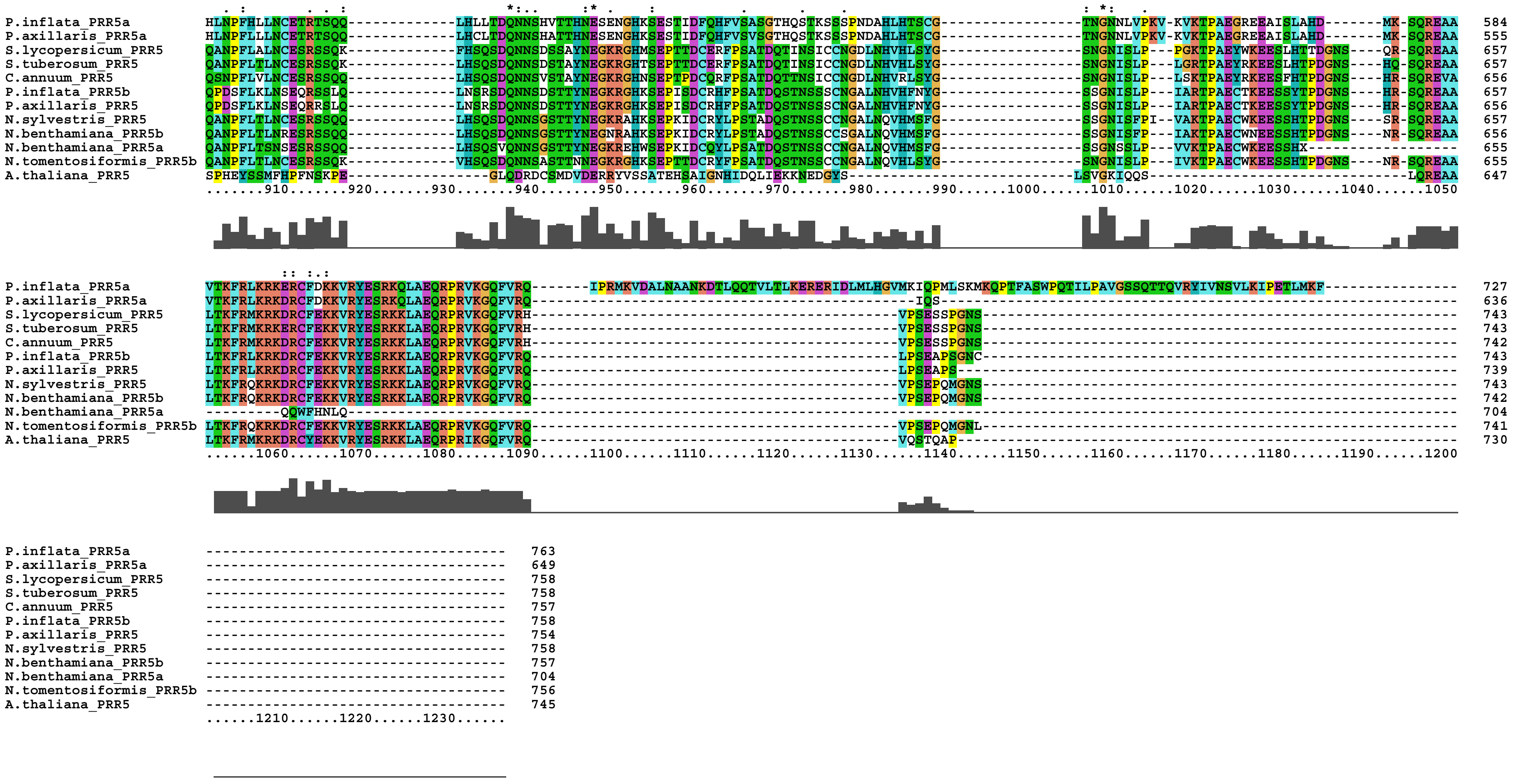
P.inflata_PRR5a 584
P.axillaris_PRR5a 555
S.lycopersicum_PRR5 657
S.tuberosum_PRR5 657
C.annuum_PRR5 656
P.inflata_PRR5b 657
P.axillaris_PRR5 656
N.sylvestris_PRR5 657
N.benthamiana_PRR5b 656
N.benthamiana_PRR5a 655
N.tomentosiformis_PRR5b 655
A.thaliana_PRR5 647
P.inflata_PRR5a 727
P.axillaris_PRR5a 636
S.lycopersicum_PRR5 743
S.tuberosum_PRR5 743
C.annuum_PRR5 742
P.inflata_PRR5b 743
P.axillaris_PRR5 739
N.sylvestris_PRR5 743
N.benthamiana_PRR5b 742
N.benthamiana_PRR5a 704
N.tomentosiformis_PRR5b 741
A.thaliana_PRR5 730
P.inflata_PRR5a 763
P.axillaris_PRR5a 649
S.lycopersicum_PRR5 758
S.tuberosum_PRR5 758
C.annuum_PRR5 757
P.inflata_PRR5b 758
P.axillaris_PRR5 754
N.sylvestris_PRR5 758
N.benthamiana_PRR5b 757
N.benthamiana_PRR5a 704
N.tomentosiformis_PRR5b 756
A.thaliana_PRR5 745

Fig. S5. Local alignment of GIGANTEA proteins. The proteins correspond to the gene models for *Arabidopsis thaliana* (AAT80910.1), Petunia *axillaris* (GI1 Peaxi162Scf00360g00418.1, GI2 Peaxi162Scf00160g01744.1) and *P. inflata* (GI1 Peinf101Scf00386g02019.1, GI2 Peinf101Scf02877g01057.1, GI3 Peinf101Scf02652g00046.1).

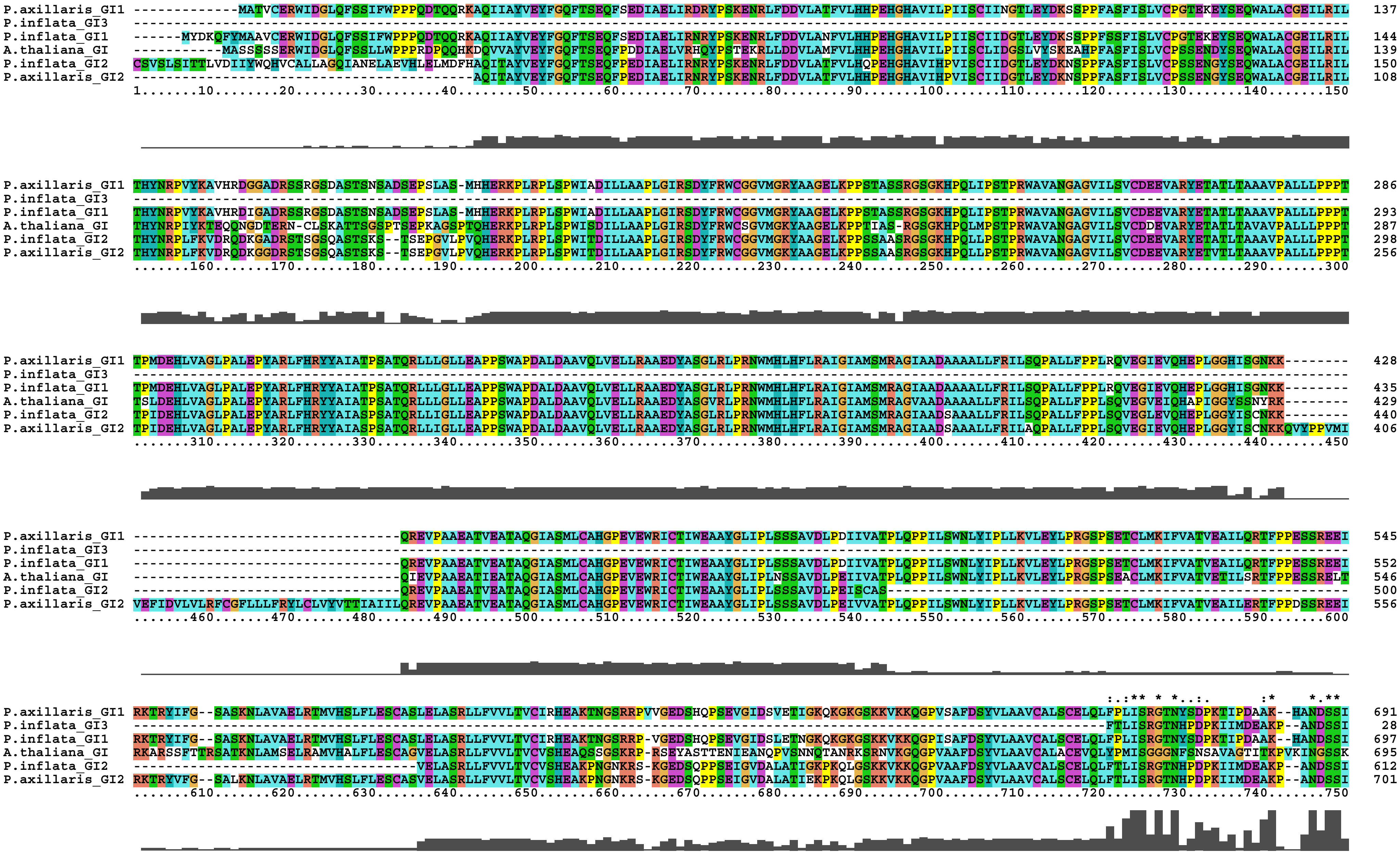

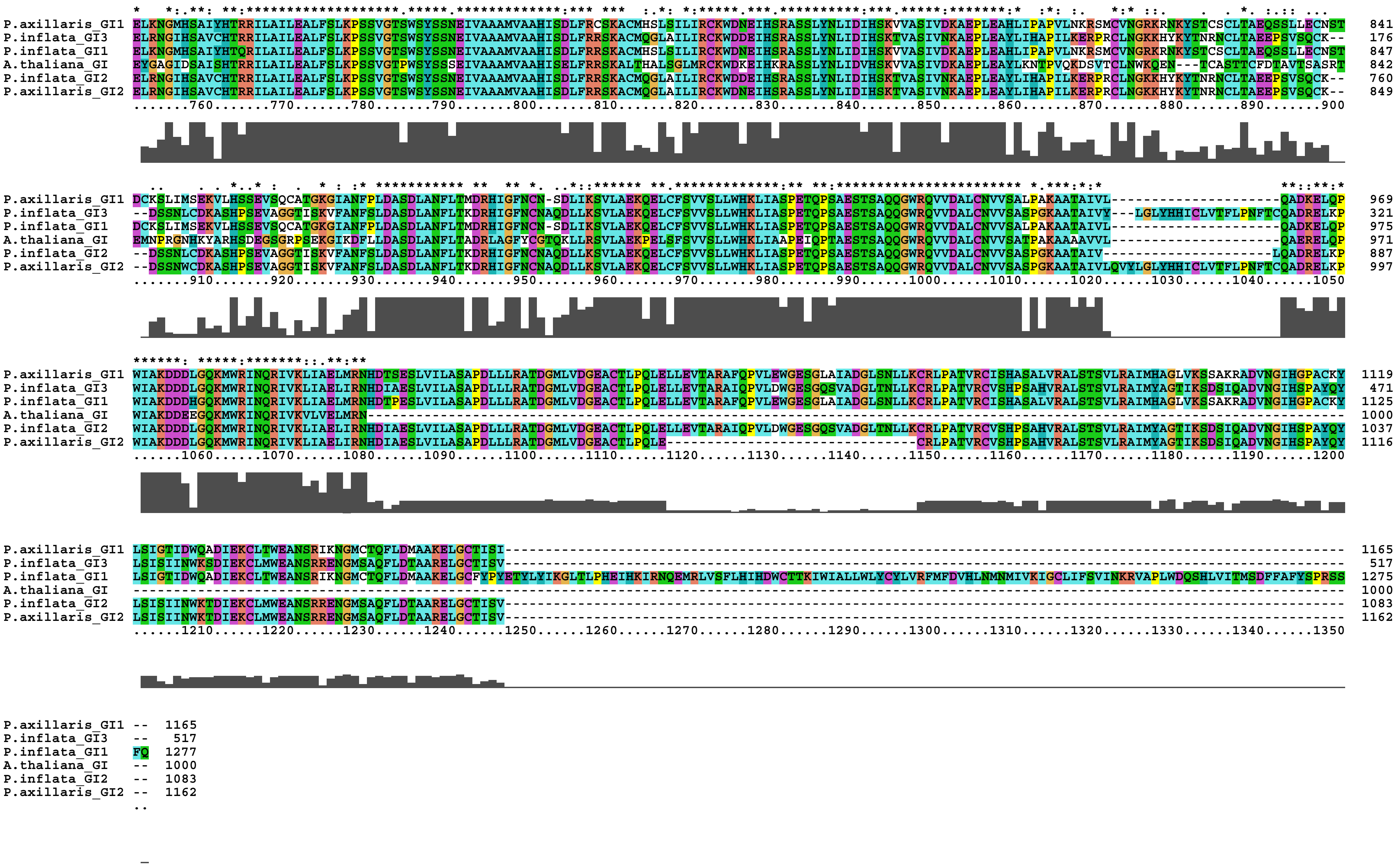

* *:.**: **:*******************.*****.*************:*** *** :.*: *:*****.***.**********:***.*****:*******:* :*: :. *:* ::. . . *. .::: ...
P.axillaris_GI1 ELKNGMHSAIYHTRRILAILEALFSLKPSSVGTSWSYSSNEIVAAAMVAAHISDLFRCSKACMHSLSILIRCKWDNEIHSRASSLYNLIDIHSKVVASIVDKAEPLEAHLIPAPVLNKRSMCVNGRKRNKYSTCSCLTAEQSSLLECNST 841
P.inflata_GI3 ELRNGIHSAVCHTRRILAILEALFSLKPSSVGTSWSYSSNEIVAAAMVAAHISDLFRRSKACMQGLAILIRCKWDDEIHSRASSLYNLIDIHSKTVASIVNKAEPLEAYLIHAPILKERPRCLNGKKHYKYTNRNCLTAEEPSVSQCK-- 176
P.inflata_GI1 ELKNGMHSAIYHTQRILAILEALFSLKPSSVGTSWSYSSNEIVAAAMVAAHISDLFRRSKACMHSLSILIRCKWDNEIHSRASSLYNLIDIHSKVVASIVDKAEPLEAHLIPAPVLNKRSMCVNGRKRNKYSTCSCLTAEQSSLLECNST 847
A.thaliana_GI EYGAGIDSAISHTRRILAILEALFSLKPSSVGTPWSYSSSEIVAAAMVAAHISELFRRSKALTHALSGLMRCKWDKEIHKRASSLYNLIDVHSKVVASIVDKAEPLEAYLKNTPVQKDSVTCLNWKQEN---TCASTTCFDTAVTSASRT 842
P.inflata_GI2 ELRNGIHSAVCHTRRILAILEALFSLKPSSVGTSWSYSSNEIVAAAMVAAHISDLFRRSKACMQGLAILIRCKWDDEIHSRASSLYNLIDIHSKTVASIVNKAEPLEAYLIHAPILKERPRCLNGKKHYKYTNRNCLTAEEPSVSQCK-- 760
P.axillaris_GI2 ELRNGIHSAVCHTRRILAILEALFSLKPSSVGTSWSYSSNEIVAAAMVAAHISDLFRRSKACMQGLAILIRCKWDNEIHSRASSLYNLIDIHSKTVASIVNKAEPLEAYLIHAPILKERPRCLNGKKHYKYTNRNCLTAEEPSVSQCK-- 849
.......760.......770.......780.......790.......800.......810.......820.......830.......840.......850.......860.......870.......880.......890.......900
.. . . *..* : . * : :* *********** ** ** *. ..*::****** **.*************:** **:************************ *.***:*:* **::**:*
P.axillaris_GI1 DCKSLIMSEKVLHSSEVSQCATGKGIANFPLDASDLANFLTMDRHIGFNCN-SDLIKSVLAEKQELCFSVVSLLWHKLIASPETQPSAESTSAQQGWRQVVDALCNVVSALPAKAATAIVL---------------------QADKELQP 969
P.inflata_GI3 --DSSNLCDKASHPSEVAGGTISKVFANFSLDASDLANFLTKDRHIGFNCNAQDLLKSVLAEKQELCFSVVSLLWHKLIASPETQPSAESTSAQQGWRQVVDALCNVVSASPGKAATAIVY---LGLYHHICLVTFLPNFTCQADRELKP 321
P.inflata_GI1 DCKSLIMSEKVLHSSEVSQCATGKGIANFPLDASDLANFLTMDRHIGFNCN-SDLIKSVLAEKQELCFSVVSLLWHKLIASPETQPSAESTSAQQGWRQVVDALCNVVSALPAKAATAIVL---------------------QADKELQP 975
A.thaliana_GI EMNPRGNHKYARHSDEGSGRPSEKGIKDFLLDASDLANFLTADRLAGFYCGTQKLLRSVLAEKPELSFSVVSLLWHKLIAAPEIQPTAESTSAQQGWRQVVDALCNVVSATPAKAAAAVVL---------------------QAERELQP 971
P.inflata_GI2 --DSSNLCDKASHPSEVAGGTISKVFANFSLDASDLANFLTKDRHIGFNCNAQDLLKSVLAEKQELCFSVVSLLWHKLIASPETQPSAESTSAQQGWRQVVDALCNVVSASPGKAATAIV---------------------LQADRELKP 887
P.axillaris_GI2 --DSSNWCDKASHPSEVAGGTISKVFANFSLDASDLANFLTKDRHIGFNCNAQDLLKSVLAEKQELCFSVVSLLWHKLIASPETQPSAESTSAQQGWRQVVDALCNVVSASPGKAATAIVLQVYLGLYHHICLVTFLPNFTCQADRELKP 997
.......910.......920.......930.......940.......950.......960.......970.......980.......990......1000......1010......1020......1030......1040......1050
******: *****:*******::.**:**
P.axillaris_GI1 WIAKDDDLGQKMWRINQRIVKLIAELMRNHDTSESLVILASAPDLLLRATDGMLVDGEACTLPQLELLEVTARAFQPVLEWGESGLAIADGLSNLLKCRLPATVRCISHASALVRALSTSVLRAIMHAGLVKSSAKRADVNGIHGPACKY 1119
P.inflata_GI3 WIAKDDDLGQKMWRINQRIVKLIAELIRNHDIAESLVILASAPDLLLRATDGMLVDGEACTLPQLELLEVTARAIQPVLDWGESGQSVADGLTNLLKCRLPATVRCVSHPSAHVRALSTSVLRAIMYAGTIKSDSIQADVNGIHSPAYQY 471
P.inflata_GI1 WIAKDDDHGQKMWRINQRIVKLIAELMRNHDTPESLVILASAPDLLLRATDGMLVDGEACTLPQLELLEVTARAFQPVLEWGESGLAIADGLSNLLKCRLPATVRCISHASALVRALSTSVLRAIMHAGLVKSSAKRADVNGIHGPACKY 1125
A.thaliana_GI WIAKDDEEGQKMWKINQRIVKVLVELMRN--- 1000
P.inflata_GI2 WIAKDDDLGQKMWRINQRIVKLIAELIRNHDIAESLVILASAPDLLLRATDGMLVDGEACTLPQLELLEVTARAIQPVLDWGESGQSVADGLTNLLKCRLPATVRCVSHPSAHVRALSTSVLRAIMYAGTIKSDSIQADVNGIHSPAYQY 1037
P.axillaris_GI2 WIAKDDDLGQKMWRINQRIVKLIAELIRNHDIAESLVILASAPDLLLRATDGMLVDGEACTLPQLE-------------------------------CRLPATVRCVSHPSAHVRALSTSVLRAIMYAGTIKSDSIQADVNGIHSPAYQY 1116
......1060......1070......1080......1090......1100......1110......1120......1130......1140......1150......1160......1170......1180......1190......1200
P.axillaris_GI1 LSIGTIDWQADIEKCLTWEANSRIKNGMCTQFLDMAAKELGCTISI-- 1165
P.inflata_GI3 LSISIINWKSDIEKCLMWEANSRRENGMSAQFLDTAARELGCTISV-- 517
P.inflata_GI1 LSIGTIDWQADIEKCLTWEANSRIKNGMCTQFLDMAAKELGCFYPYETYLYIKGLTLPHEIHKIRNQEMRLVSFLHIHDWCTTKIWIALLWLYCYLVRFMFDVHLNMNMIVKIGCLIFSVINKRVAPLWDQSHLVITMSDFFAFYSPRSS 1275
A.thaliana_GI -- 1000
P.inflata_GI2 LSISIINWKTDIEKCLMWEANSRRENGMSAQFLDTAARELGCTISV-- 1083
P.axillaris_GI2 LSISIINWKTDIEKCLMWEANSRRENGMSAQFLDTAARELGCTISV-- 1162
......1210......1220......1230......1240......1250......1260......1270......1280......1290......1300......1310......1320......1330......1340......1350
P.axillaris_GI1 -- 1165
P.inflata_GI3 -- 517
P.inflata_GI1 FQ 1277
A.thaliana_GI -- 1000
P.inflata_GI2 -- 1083
P.axillaris_GI2 -- 1162
..

Fig. S6. Normalized expression (NE) for the biological replicates. Expression of clock genes and associated-genes in leaves (red dots) and petals (blue dots) under a 12 light:dark (LD) and constant darkness (DD). Gene expression was normalized to *PhACT*, the value of every single biological replicate is showed here. Grey areas indicate dark periods. ZT denotes *zeitgeber* time.

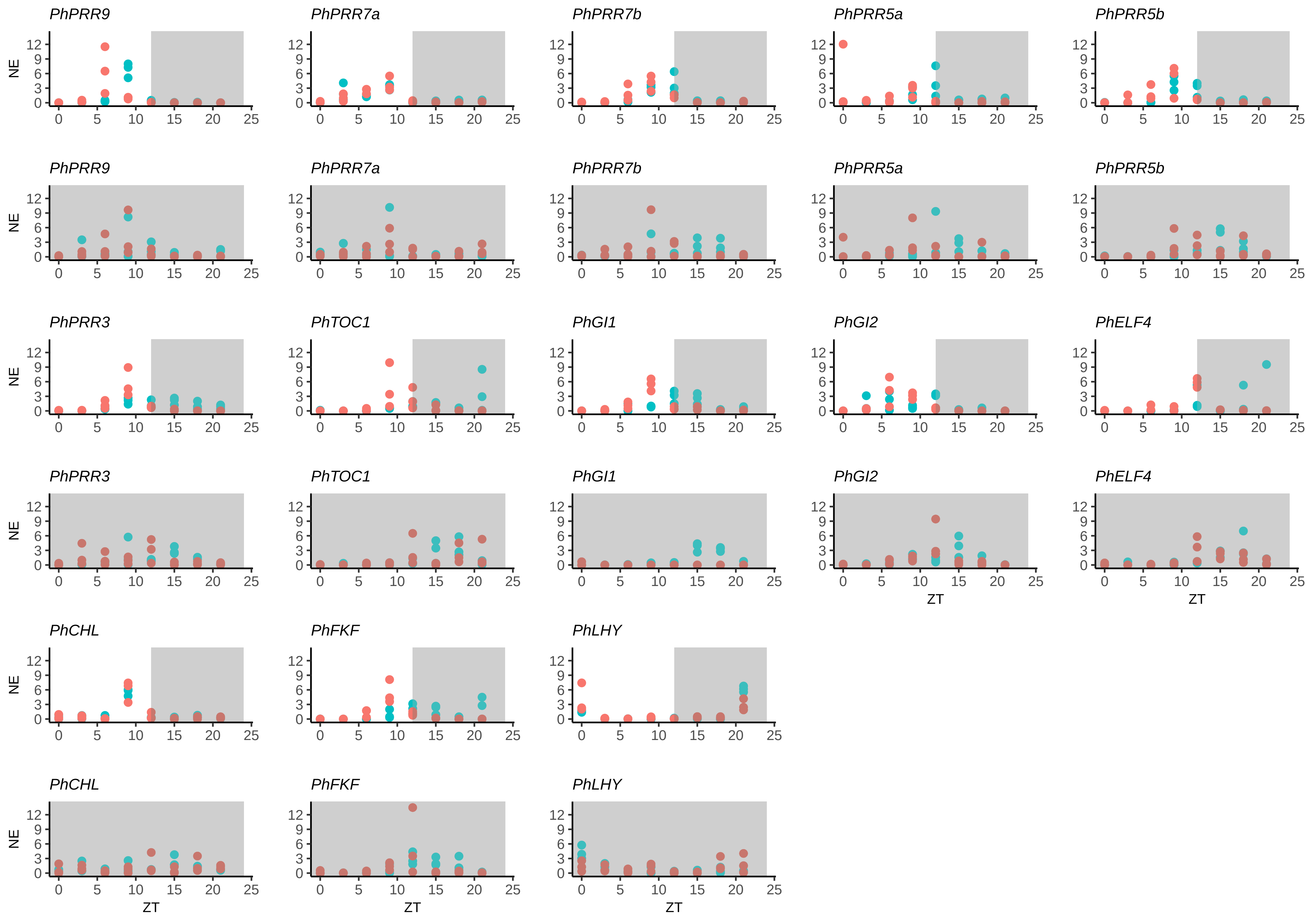
PhPRR9
PhPRR7a
PhPRR7b
PhPRR5a
PhPRR5b
PhPRR9
PhPRR7a
PhPRR7b
PhPRR5a
PhPRR5b
PhPRR3
PhTOC1
PhGI1
PhGI2
PhELF4
PhPRR3
PhTOC1
PhGI1
PhGI2
PhELF4
PhCHL
PhFKF
PhLHY
PhCHL
PhFKF
PhLHY
NE
ZT

Table S1 PSEUDO-RESPONSE REGULATORs (PRRs) protein accessions used in the phylogenetic reconstruction and for the annotation of protein sequences.

Species	Protein	Accession
Antirrhinum majus	PRR5	Am07g14220
	PRR7	Am07g35120
	TOC1	Am01g23590
Arabidopsis thaliana	PRR3	AT5G60100.2
	PRR5	NP_568446.2
	PRR7	AT5G02810.1
	PRR9	AT2G46790
	TOC1	AT5G61380.1
Amborella trichopoda	PRR37	XP_006828277.1
	PRR73	XP_020529326.1
	PRR9	XP_011629028.1
	TOC1	XP_011625651.1
Capsicum annuum	PRR5	CA03g21230
	PRR9	CA10g05550
	PRR7	CA10g11870
	TOC1	CA03g29830
Nicotiana attenuata	TOC1	XP19229967
N. benthamiana	PRR3a	Niben101Scf05009g04012.1
	PRR5a	Niben101Scf00225g05002.1
	PRR5b	Niben101Scf01109g04021.1
	PRR7a	Niben101Scf00294g03009.1
	PRR7b	Niben101Scf01994g01007.1
	PRR9a	Niben101Scf11670g02004.1
	PRR9b	Niben101Scf02026g04007.1
	TOC1a	Niben101Scf11469g00017.1
	TOC1b	Niben101Scf00063g06009.1
N. sylvestris	PRR3	gi|698444565|
	PRR5	gi|698515802|
	PRR7	gi|698585162|
	TOC1	XP_009791525.1
N. tomentosiformis	PRR3a	gi|697159824|
	PRR5a	XP_018627531.1
	PRR5b	gi|697192415|
	PRR7	gi|697100515|
	PRR9	mRNA_36525_cds
	TOC1	gi|697126282|
Petunia axillaris	PRR37	Peaxi162Scf01024g00009.1
	PRR5b	Peaxi162Scf00912g00022.1
	PRR5a	Peaxi162Scf00542g03011.1
	PRR7a	Peaxi162Scf01517g00133.1
	PRR7b	Peaxi162Scf00146g01328.1
	PRR9	Peaxi162Scf00313g00210.1
	TOC1	Peaxi12Scf00274g00351.1
P. inflata	PRR3	Peinf101Scf02768g00025.1
	PRR5a	Peinf101Scf00223g03002.1
	PRR5b	Peinf101Scf01482g12029.1
	PRR7a	Peinf101Scf00931g05033.1

	PRR7b	Peinf101Scf00756g02019.1
	PRR9	Peinf101Scf00559g10011.1
	TOC1	Peinf101Scf01556g09043.1
Physcomitrella patens	PRR37	XP_024364994.1
Solanum lycopersicum	PRR3	Solyc04g049670.2
	PRR5	Solyc03g081240.2
	PRR7	Solyc10g086000.1
	PRR9	Solyc10g005030.2
	TOC1	Solyc03g115770.2
S. tuberosum	PRR5	PGSC0003DMP400001175
	PRR7	PGSC0003DMP400001175
	PRR9	XP_006352628
Vitis vinifera	TOC1	GSVIVG01007965001
	PRR3	GSVIVT01025088001
	PRR5	GSVIVG01027443001
	PRR7	GSVIVG01032644001
	PRR9	GSVIVT01038647001

Table S2 GIGANTEA (GI) protein accessions used in the phylogenetic reconstruction.

Species	Protein	Accession
Arabidopsis thaliana	GI	AAT80910.1
Amborella trichopoda	GI	PRGDB24499
Antirrhinum majus	GI	Am06g07460.P03
Brachypodium distachyon	GI	XP_003564357.1
Cicer arietinum	GI1	XP_004496435.1
	GI2	XP_004496436.1
Fragaria vesca	GI	XP_004290483.1
Glycine max	GI1	BAJ22595.1
	GI2	BAN82589.1
	GI3	ACA24490.1
Marchantia polymorpha	GI	Mapoly0019s0145.1
Medicago truncatula	GI	XP_003592048.2
Nicotiana benthamiana	GI1	NbS00010804g0011.1
	GI2	NbS00006512g0008.1
	GI3	Niben101Scf09649g01002.1
	GI4	NbS00010805g0006.1
Oryza sativa	GI	XP_015649578.1
Panicum hallii	GI	Pahal.E04224.2
Petunia axillaris	GI1	Peaxi162Scf00360g00418.1
	GI2	Peaxi162Scf00160g01744.1
P.inflata	GI1	Peinf101Scf00386g02019.1
	GI2	Peinf101Scf02877g01057.1
	GI3	Peinf101Scf02652g00046.1
Pisum sativum	GI	ABP81863.1
Selaginella moellendorffii	GI	XP_024528715.1
Setaria italica	GI	Seita.5G129500.1
S.viridis	GI	Sevir.5G127000.1
Solanum lycopersicum	GI1	Solyc04g071990.2.1
	GI2	Solyc12g056650.1.1
S.tuberosum	GI	XP_006359039.1
Triticum aestivum	GI3	AAT79487.1
Vigna radiata	GI	XP_014514153.1
V.unguiculata	GI	Vun_T01130.1_6
Vitis vinifera	GI	XP_010665061.1
Zea mays	GI1	GRMZM2G107101_T03
	GI2	GRMZM5G844173_T01

Table S3 Primers used for qPCR.

Gene	Forward primer	Reverse primer
PhCHL	TGCATCTGTTGGCTCTGTTT	CCCCAACCCAATCTCTTAGC
PhFKF	CTGGGCAACCTCCAAAGTT	CATGGATCAGAATCTTGTTTGCT
PhGI1	TGGAGAAAGGGCAGAGACAT	GTGGAGCCACCCTTACGTT
PhGI2	TTTAGAGTCCTTTCACTCATCCATC	AATACAGCATTTGTTACATGGAGGT
PhLHY	CGACGTGGTAGGAATTGCATC	GCAAGAAATGGTCATCAAAGGAC
PhPRR3	GTGGAAGTGGTGATCGCTCT	TCCATCCTGACGCACAAAT
PhPRR5a	CCAATGATGCCCATCTTCAT	ACGCTGCTCTGCAAGTTGTT
PhPRR5b	GGAACTCCCATCGATCTCAA	GGAGCCTCACTAGGTAATTGACG
PhPRR7a	CAGAAGAGGAAGGAGCGATG	AGGGAAAGTAGGAAGGAAAGCA
PhPRR7b	AGGTGCAAATGCGGAAAGT	AGCATCTATCTTTCTTCTTGTGACG
PhPRR9	GAGCTCATGTTTCCGAGAGC	GGTCGCTGTTCTGCAAGTCT
PhTOC1	TGATGGTAAGGGGAGCAAAG	CTGAAGCAGGATGCCCATTA
PhACT	TGCACTCCCACATGCTATCCT	TCAGCCGAAGTGGTGAAAGAG
